# Strategic Approaches for Co-Encapsulation of Bioactive Compounds: Technological Advances and Mechanistic Insight

**DOI:** 10.3390/foods14122024

**Published:** 2025-06-07

**Authors:** Chaoting Wen, Jialuo Tang, Liyan Cao, Meidi Fan, Xinying Lin, Guoyan Liu, Li Liang, Xiaofang Liu, Jixian Zhang, Youdong Li, Xin Xu

**Affiliations:** 1 College of Food Science and Engineering, Yangzhou University, Yangzhou 225127, China; chaoting@yzu.edu.cn (C.W.); 18469510402@163.com (J.T.); mz120222071@stu.yzu.edu.cn (L.C.); 15155725798@163.com (M.F.); lin1224in@163.com (X.L.); yzufsff@163.com (G.L.); liangli0508@hotmail.com (L.L.); 008040@yzu.edu.cn (Y.L.); 2 School of Tourism and Cuisine, Yangzhou University, Yangzhou 225127, China; liuxf@yzu.edu.cn

**Keywords:** co-delivery carriers, bioactive substances, characterization technique, release mechanisms, molecular docking

## Abstract

In food science, the application of bioactive compounds is promising, but the deficiencies in their solubility, stability, and bioavailability seriously limit their efficacy. Co-delivery can control the release of bioactive substances and achieve synergistic effects to enhance efficacy. For this reason, co-delivery systems have emerged as a novel strategy that has attracted much attention from researchers in recent years. This review provides an in-depth analysis of the unique properties of different bioactive ingredients and systematically describes the latest research progress of co-encapsulation systems such as emulsions, nanoparticles, and liposomes. It also introduces their advanced characterization tools. Meanwhile, the kinetic law of the release of bioactive ingredients in vivo and its molecular mechanism are discussed in depth through experimental data and theoretical analysis. In addition, the potential application value of the co-encapsulation system in the food industry is also introduced in detail. In summary, this study not only provides a solid theoretical foundation and rich reference basis for further research in the field of co-encapsulation, but also provides new ideas and directions to promote the construction and development of rational and efficient co-encapsulation systems, and to promote the innovation and development of functional foods and precision nutrition.

## 1. Introduction

With the rapid development of food technology and the continuous increase in consumption level, people are paying more attention to daily dietary health as well as disease prevention, and the demand for functional foods is increasing. This significant shift reflects the consumer’s strong demand for disease prevention through daily dietary interventions. Bioactive substances, such as polyphenols, drugs, vitamins, and proteins, not only have anti-inflammatory and antioxidant abilities and the ability to regulate intestinal flora [1], but also have a variety of pharmacological effects, such as anticancer, anticardiovascular disease, anti-obesity, anti-aging, and immune regulation effects [2,3], which play an important role in functional foods. However, these bioactives face many challenges in practical applications. Low water solubility (lipid-soluble nutrients) or environmental sensitivity (UV, pH, heat, gastrointestinal) can lead to reduced stability or even loss of bioactivity, which ultimately affects their absorption and utilization by the human body and reduces bioavailability [4,5,6,7]. To overcome these limitations, encapsulation technology has emerged as an effective solution. Currently, researchers have developed a variety of delivery systems, such as emulsions, nanoparticles, hydrogels, microcapsules, and nanoliposomes, for loading various functional ingredients. These delivery systems are capable of protecting bioactives from damage by external factors during food storage, processing, and in the gastrointestinal tract [8].

However, these deliveries have primarily focused on the encapsulation of single bioactive compounds. In recent years, co-delivery systems have also received more attention. The coexistence of multiple bioactives in a delivery system not only enhances their stability and bioavailability but also the synergistic effects of these actives compared to the ingestion of a single active substance [9]. Researchers have used delivery vehicles to embed multiple bioactives simultaneously and applied them in the field of functional foods. Delivery systems such as emulsions, nanoparticles, and nanoliposomes have been widely used in the co-delivery of bioactives, mainly because of their biocompatibility, which allows them to encapsulate multiple bioactives simultaneously. In addition, these delivery systems can control the release of bioactives in vivo, thus improving their bioavailability [10,11], providing new ideas and methods for the development of functional foods. Based on the synergistic effect of nutrients, co-delivery technology significantly enhances the physiological efficacy of functional foods by improving bioavailability and targeted delivery efficiency. Studies have shown that such technologies can enable the synergistic effects of multi-nutrients in the areas of immunomodulation, blood pressure homeostasis, and sleep improvement, providing a safer “green alternative” to traditional drugs [12]. In addition, the combined action delivery system has attracted attention in the field of freshness preservation. Freeze-dried mushroom particles co-loaded with curcumin and quercetin were applied to cooked beef patties with lower thiobarbituric acid content than single-substance-encapsulated and physically mixed systems with the same concentration, effectively inhibiting oxidation and extending shelf life [13].

To better construct the co-delivery carrier system, this study used Web of Science, PubMed, Google Scholar, and X-MOL databases to search for articles. Titles, abstracts, or keywords containing terms related to “delivery system” and “co-embedding” were selected for detailed analysis. In addition to descriptors, other inclusion criteria included articles published between January 2004 and May 2025 in any language. The non-academic literature (e.g., news articles, blog posts) and the literature on topics not directly related to the delivery field were excluded. The research presented in this manuscript encompasses several key aspects: (1) The solubility, structure, morphology, bioactivity, defects, and improvement effects of co-delivery carriers encapsulating various types of bioactive substances were concluded. (2) The main types of co-delivery carriers, specialized and general characterization tools for characterizing different carriers, were broadly characterized. (3) The gastrointestinal delivery kinetics of co-delivery carriers and their mechanisms of delivery of bioactive substances were emphasized. (4) The applications of co-delivery in different fields were summarized. This study can provide some theoretical references for the field of co-delivery and promote the development of rational and efficient co-delivery system construction.

## 2. Types of Co-Delivered Active Substances

Polyphenols, the natural compounds that have received the most attention for their antioxidant and anti-inflammatory functions, are chemically unstable and have low bioavailability [1], making them a typical target for delivery system research. Secondly, probiotics are living biological agents that require stringent delivery conditions, and research has focused on improving their delivery efficiency and intestinal colonization ability [14]. Again, vitamins cover both water-soluble and fat-soluble categories, with significant differences in delivery strategies [15]. Therefore, this review discusses bioactives by categorizing them into these three main groups. This paper summarizes the structure, properties, functions, and stability of bioactives as shown in Table 1. Polyphenols are recognized for their potent antioxidant and anticancer properties [16]. Additionally, probiotics provide a variety of health benefits through their biological mechanisms in the body [14], while vitamins are essential for supporting various metabolic processes [15]. However, when taken in their free form, these compounds often become unstable in the body, degrading during digestion and liver processing, which reduces their effectiveness. To tackle these issues, researchers are focused on creating advanced delivery systems that protect these bioactive compounds and enable controlled release. This is particularly important for precision nutrition. Co-delivery platforms, which encapsulate multiple bioactive compounds together, show promise for enhancing their combined effectiveness and addressing complex nutritional needs. A co-delivery system that delivers multiple active ingredients simultaneously and combines them into a single system can effectively improve the function of substances and enhance efficacy through complementary effects [17]. Specifically, it can reduce the potential side effects of high concentrations of a single substance by analogizing the barrier effect and increasing the amount of active ingredients loaded into the delivery material. In addition, a co-delivery system can control the ratio of active substances to personalize the content of different nutrients. Co-delivery significantly improves therapeutic efficacy and safety through synergistic effects, complementary stability, and functional integration [18]. Co-delivery platforms that encapsulate multiple bioactive compounds together are expected to increase the combined efficacy of these compounds and address complex nutritional needs and diseases.

### 2.1. Polyphenols

Polyphenols are naturally occurring chemical components of vegetables, fruits, grains, and beverages. The antioxidant activity of polyphenols is closely related to their core structural features. Polyphenols mainly exhibit two basic backbone structures, the C6-C1 type and the C6-C3-C6 type [40]. They comprise one or more aromatic ring structures with single or multiple hydroxyl (OH) groups bound to them. Based on this structural property, it gives them the ability to scavenge free radicals, chelate metal ions, and provide hydrogen atoms or electrons, thus effectively blocking the oxidative chain reaction [41]. Based on chemical structures, natural polyphenols can be divided into five classes, including flavonoids, phenolic acids, lignans, stilbenes, and other polyphenols. Flavonoids and phenolic acids account for about 60% and 30% of all natural polyphenols, respectively [42]. Natural polyphenols possess a variety of biological activities, such as antioxidant [43,44], anti-inflammatory [45,46], and anticancer [47,48] activities, among others. It is worth noting that the antioxidant efficacy of polyphenols is significantly correlated with the number and spatial arrangement of hydroxyl groups on their benzene rings, and an increase in the degree of hydroxyl substitution enhances the electron delocalization effect, as gallic acid shows strong antioxidant activity due to its neighboring trihydroxyl structure. Based on the hydrophilic and hydrophobic properties of polyphenols, common natural polyphenols can be classified as hydrophilic polyphenols ((−)-epigallocatechin-3-gallate (EGCG) and Anthocyanins (ACNs)) and hydrophobic polyphenols (curcumin (Cur) and resveratrol (Res)).

EGCG, a hydrophilic polyphenol (potent catechin), is widely found in green tea. EGCG is the most active ingredient of tea polyphenols. Tea polyphenols are widely applied in the beverage and other food industries, so the co-delivery system of EGCG shows great potential for application in the beverage industry [49]. EGCG is rich in phenolic hydroxyl groups, which have strong antioxidant activity, anticancer activity, and cardiovascular disease prevention, among other effects [25]. Furthermore, previous studies have also confirmed that EGCG suppresses PPARγ activity and inhibits the proliferation of preadipocytes by preventing the differentiation process into mature adipocytes synergistically, thus reducing adipogenesis [35]. EGCG is a water-soluble component, but it is unstable under neutral or alkaline conditions and is prone to degradation into a yellow-brown substance [49]. In addition, the low bioavailability, poor digestive stability, and poor intestinal absorption capacity of EGCG leads to sensitivity towards pH, light, oxygen, temperature, and ionic strength. The free EGCG is absorbed in the stomach and in the early stage of the small intestine, with low oral bioavailability due to efflux and pre-degradation [35]. ECCG can synergistically exert antioxidant, antitumor, anti-inflammatory, and other effects with many bioactive substances, such as Res, Cur, and quercetin [50,51]. Cur and EGCG are co-embedded in W/O/W emulsions, which can inhibit the proliferation of PC3 cells by up-regulating the expression of p21, and then play a synergistic role in increasing the anticancer effect [52,53]. Therefore, it is necessary to construct a delivery system to simultaneously deliver EGCG and other active substances. The co-delivery system could effectively enhance the stability of EGCG and other active substances, avoid their degradation during gastric digestion, promote their release and absorption in the intestine, and thereby improve the bioavailability of active substances. Nanoparticle-stabilized Pickering emulsions are common carriers for the delivery of EGCG and other bioactive substances. Liu et al. [49] used the Maillard reaction to construct Pickering emulsions co-delivering curcumin and EGCG, and their storage and thermal stability were evaluated. The results of this study showed that the Pickering emulsions effectively protected the bioactive substances from harmful conditions (such as acid, heat, and salt ion conditions). Moreover, the emulsion structures showed no significant differences after heating at 85 °C for 30 min, with a relatively uniform oil droplet size distribution and no significant increase in the average droplet size. In addition, the droplet size of the emulsions did not change significantly during 30 days of storage, which effectively slowed down the degradation of the active substance. For example, the complex formed by tea polyphenols (EGCG) and D-alpha-tocopherol (VE) exhibits synergistic milk-breaking and fat-targeting functions in obesity treatment. The PmFL microspheres developed by Wu et al. [54] achieved fat excretion efficiencies in the gastrointestinal tract ranging from 63.10% to 81.13% by integrating EGCG and VE, which were significantly higher than that of a single component. In addition, the microspheres reduced the weight gain of high-fat-diet rats by 17.02%, close to the level of the normal diet group, showing the metabolic regulation advantages of the polyphenol combination. In the study of Wang et al. [35], the Pickering double emulsion co-delivering EGCG and lycopene was of great physical stability and high absorption efficiency in the small intestine, which enhanced the bio-accessibility of the two bioactive substances. Moreover, when EGCG and LYC were co-delivered by Pickering double emulsion, the AMPK pathway in the emulsion was activated to achieve a synergistic lipid-lowering effect. In previous studies, gel delivery systems could effectively enhance the enteral phase controlled release and bio-accessibility of EGCG. The emulsion gel prepared by Chen et al. [55] increased the bio-accessibility of EGCG from 30 to 50%. The structures of bigels prepared by Lu et al. [56] could be well modulated by the oleogelator, which then affected the release of the functional ingredients incorporated, which was beneficial for the development of functional foods.

ACNs (water-soluble polyphenolic flavonoids) are natural plant colorants found in fruits and vegetables with biological activities such as antioxidant, anti-inflammatory, anti-aging, and anticancer activities [57]. However, ACNs are susceptible to degradation in light, oxygen, pH > 7, and temperature > 50 °C, and are poorly stabilized during digestion due to enzyme and pH changes, resulting in gastrointestinal absorption losses [58]. Consequently, how to improve the stability and bio-accessibility of ACNs was the focus of the research, and the rational design of delivery vehicles was an important method to protect ACNs. Nanoliposomes and microencapsulated co-delivery vehicles were designed to co-encapsulate lipo-soluble bioactive substances and water-soluble ACNs, thereby improving the antioxidant properties and stability of ACNs and preventing premature release of ACNs during gastrointestinal digestion. Eloá Lourenço do Carmo et al. [59] characterized the microparticles obtained from the co-encapsulation of ACNs extracted from red grape skins and α-tocopherol via spray drying. They found that the antioxidant activity of the carrier effectively increases to 88%, and the total phenolic content is increased to 8.69 mg GAE/g powder, confirming the synergistic effect of co-encapsulation to enhance the bioactivities. In the study by Xu et al. [60], novel liposomes co-encapsulating Docosahexaenoic acid (DHA) and ACNs were applied to infant milk powder, and their absorption and transport mechanisms in the infant gastrointestinal tract were investigated. The co-encapsulation strategy effectively improved the stability and organoleptic acceptability of the ACNs, and the microencapsulation effectively enhanced the storage stability and bio-accessibility of the ACNs to achieve the targeted release of the active substances. The entrapment of liposomes and DHA promoted the absorption of ACNs by Caco-2 cells. Furthermore, liposomes had the antioxidant capacity to reduce ROS levels after entering Caco-2 cells. Co-pigmentation is an effective method of enhancing the stability of anthocyanins by combining an electron-rich π-system containing a co-pigment with an electron-deficient flavoalkyl cation to form a complex. Moshfegh et al. investigated the copolymerization of sour cherry anthocyanins with tannic acid, followed by encapsulation. It was shown that the use of tannic acid as a co-pigment resulted in higher anthocyanin retention and redder color [61].

Cur is a hydrophobic polyphenol extracted from turmeric [9,62]. Many in vivo and in vitro experiments have revealed that Cur possesses a variety of biological activities, including antioxidant [63], intestinal disease prevention [64], anticancer [32], and antimicrobial effects [65,66]. Based on the rich biological activity of Cur, it shows great potential for application in the fields of personalized nutraceutical carriers, functional foods, and dietary supplements [21,65,67]. However, Cur has the shortcomings of poor water solubility (about 11 ng/mL), low stability (sensitive to light, heat, and oxygen, and prone to degradation), low bioavailability (about 1%; fast intestinal metabolism), poor permeability, and poor targeting [68]. These deficiencies made Cur unable to give full expression to its functional properties, and it was difficult to apply to the food system, reducing its usage as a therapeutic drug [69]. Many studies have proved that the combination of Cur and Res could achieve synergistic effects. Additionally, it was reported that Cur and Res are preferentially located in the hydrophobic acyl-chain region and outer headgroup region facing the water subphase, respectively, both of which could rigidify the liposomal membrane [70]. Res is also a lipo-soluble polyphenol extracted from the grape skin, is recognized as the best-known compound of the stilbene group, which has several functional activities like anticancer, anti-obesity, and antioxidant [32,71], and benefits against cardiovascular disease, cancer, and liver diseases. Res also has poor water solubility, light and heat stability, and low bioavailability. Having various benefits on their own, Cur and Res presented significant therapeutic benefits, especially for the prevention and treatment of different types of cancers through several molecular mechanisms when co-encapsulated in oleogel [65]. Thus, many researchers have extensively developed co-delivery carriers encapsulating Cur and Res to avoid the above-mentioned problems limiting the application of Cur and to improve the carrier delivery performance and bio-accessibility. The encapsulation efficiency of the two bioactive substances in nanocapsules prepared by K. Coradini et al. was close to 100%. Nano-encapsulation improved the photostability of Res and Cur, and co-loading increased the antioxidant activity of polyphenols [63]. Likewise, the microparticles co-encapsulated with Cur and resveratrol showed good release behavior and conformed to the global slower, controlled release profile (Weibull model) [32]. Guo et al. [71] prepared protein-polysaccharide-surfactant ternary complexes that co-encapsulate Cur and resveratrol through hydrogen bonding, hydrophobic interaction, and electrostatic attraction. The complex was more effective in slowing the light and thermal degradation of both nutraceuticals, as well as providing a protective effect under gastric conditions. Furthermore, it could control the release of both nutraceuticals in the intestinal phase. Cur and resveratrol in MCT-based oleogel showed significantly (*p* < 0.05) higher bio-accessibility at 50.08 ± 1.27% and 89.19 ± 0.89% under the in vitro digestion study, respectively, which contributed to 1.13-fold and 1.20-fold enhancement [65]. K. Coradini et al. [72] showed that lipid-core nanocapsules containing Res and Cur provided the most pronounced benefits compared to individually loaded polyphenols. The potentialities of this formulation were demonstrated by the improvement in antioedematogenic activity and the attenuation of cartilage and bone damage.

Co-delivery carriers significantly enhance the physical and gastrointestinal stability of polyphenols (EGCG, Que, Cur, Res, etc.), but the co-delivery system has been less studied in lipid-soluble vitamins, certain phytochemicals, and combinations of different polyphenol species. The metabolic processes and release behaviors of co-delivery systems in vivo are not well studied in the existing studies, making it difficult to achieve precise regulation. In the future, smart-responsive nanocarriers can be developed to realize the precise targeted delivery of active substances. In addition, the metabolic process of the co-delivery system in vivo can be studied in depth by combining it with cross-omics research methods. It is also possible to optimize the production process and reduce the cost of complex processes (e.g., microfluidics) to promote the industrialization of co-delivery systems.

### 2.2. Probiotics

The oral administration of live probiotics has been suggested, with numerous beneficial effects for several conditions, including certain infectious disorders, diarrheal illnesses, some inflammatory bowel diseases, and, most recently, irritable bowel syndrome. Though, the delivery of such viable bacteria to the host intestine is a major challenge due to the poor survival of the ingested probiotic bacteria during the gastric transit, especially within the stomach, where the pH is highly acidic [73]. The survival of probiotics in bilayer microcapsules prepared by Ma et al. was increased by 62.85%, and the growth of probiotics during storage was further enhanced (about 3.14 times) [29]. The combination of cheese whey protein (CW), fructo-oligosaccharides (FOS), and xanthan gum (XG) demonstrates high encapsulation efficiencies of Lacticaseibacillus casei CSL3 (approximately 99%) [74]. Probiotics are live microorganisms that, when ingested in sufficient quantities, can provide health benefits to the host. In recent years, studies have shown that co-embedding probiotics with other bioactives (e.g., polyphenols, fish oil, etc.) can significantly enhance the activity, stability, and efficacy of both [75]. Holkem et al. [76] prepared microcapsules co-encapsulating Bifidobacterium animalis Lactobacillus subsp. Lactis (BLC1) and proanthocyanidin-rich cinnamon extract (PRCE), and the addition of BLC1 reduced the rate of necrotic and apoptotic cell death. Co-microencapsulation allowed for the delivery of BLC1 and PRCE into the human intestinal system with less impact on functional properties and the prevention of cancer at an early stage. Cai et al. [77] developed 3D-printed custard creams with a blend of probiotics, EGCG, and Res. The addition of EGCG increased the encapsulation efficiency of Lactobacillus plantarum by about 11%. Zhang et al. [78] constructed a cactus natural polyphenol metal framework structure-reinforced cactus polysaccharide microgel delivery system, which more effectively enhanced the gastrointestinal tolerance and colonization rate of probiotics, and the natural polyphenol, as a prebiotic cactus polysaccharide, promotes the production of short-chain fatty acids, and all three synergistically modulated the intestinal and hepatic axes, thereby alleviating acute liver injury and intestinal dysbiosis in mice.

Despite the remarkable progress of probiotic drug delivery systems in protecting strain activity and improving intestinal colonization, they still face multiple challenges in clinical application and industrialization. First, insufficient tolerance and survival in the gastrointestinal environment are the core bottlenecks. Most probiotics have low survival rates in gastric acid (pH 2.0–3.0) and bile salt environments [79]. For example, although the spray-drying process improves storage stability, high temperatures may lead to reduced survival of probiotics. In addition, the lack of targeted delivery precision to match controlled release limits efficacy. Some carriers have low release efficiencies under colon-neutral conditions that do not accurately meet the needs of probiotic colonization. In addition, synergistic mechanisms of co-delivery systems are lagging. Although studies have attempted to combine probiotics with antibiotics and prebiotics, insufficient attention has been paid to the synchronization of the release kinetics of the different active ingredients.

### 2.3. Vitamins

Vitamins play an important role in human health and are considered essential for life processes in living organisms. The reduced level of vitamins in food complexes and less absorption in the intestines often leads to vitamin deficiency in the body. Therefore, the vitamins are orally administered to the human body through fortification in foods. But the main problem is that they undergo degradation by the external environment and gastrointestinal conditions, and also possess poor bioavailability and lack necessary robustness when consumed in their pure vitamin forms [80]. Therefore, it is necessary to construct a co-delivery system, whereby multivitamins can be supplemented at the same time to provide comprehensive nutritional supplementation, and co-delivery carriers can also provide effective protection for vitamins to improve the stability and bio-accessibility of vitamins. α-Tocopherol, as a lipophilic antioxidant, can delay or prevent chronic diseases associated with oxidative stress. This vitamin can be used as an antioxidant in the food matrix to increase the shelf life of the product [59,81]. Sun et al. successfully prepared the liposome-stabilized Pickering emulsions to achieve the co-encapsulation of multiple nutrients. The emulsions stabilized by liposomes maintain stability under different conditions (pH, ionic strength, storage time, and temperature). In addition, the liposomes inhibit lipolysis in the emulsions, which further increases the bio-accessibility of vitamin B2, vitamin E, and β-carotene [82]. Sodium caseinate-stabilized co-encapsulated α-tocopherol and resveratrol O/W emulsions increase the bio-accessibility of α-tocopherol and resveratrol to 40% and 90%, respectively [7]. Many researchers have co-encapsulated Vitamin C and β-carotene to study the improvement of antioxidant properties and stability of the two actives by co-delivery carriers. Liu et al. and Hamadou et al. prepared liposome co-encapsulation of Vitamin C and β-carotene, which effectively improved the storage stability and antioxidant properties and achieved controlled release of the active substances [83,84]. The encapsulation efficiency of W/O/W emulsions co-encapsulated with vitamin C and β-carotene is more than 87% and 99%, respectively, and still can maintain around 50% retention of the antioxidant capacity after storage for 28 days at 4 °C [31].

Vitamin co-delivery systems have significantly improved the stability and bioavailability of fat- and water-soluble vitamins through carrier technologies such as liposomes, nanoparticles, and emulsions. However, existing carriers still have limitations in terms of targeted delivery precision and controlled release matching, especially in terms of the release kinetic synchronization of vitamin combinations that need to be optimized. For example, despite the potential synergistic role of vitamins K2 and D3 in the regulation of calcium metabolism, the study of their combined delivery system is still a blank field. The development of folate–iron co-delivery systems for special populations (e.g., pregnant women) is lagging, making it difficult to balance micronutrient dosage with toxicity risk. In addition, the freeze-drying of complex carriers is costly, and photosensitive vitamins (e.g., D3) still face the risk of photo-oxidative degradation in nanocarriers, which is protected by inert gas packaging, significantly increasing the economic and technical thresholds for large-scale production.

### 2.4. Others

Coenzyme Q10 (CoQ10) is a vitamin-like, oil-soluble active compound. CoQ10 functions as a well-known electron carrier in the electron transport chain, thus participating in aerobic cellular respiration; the reduced form of CoQ10 is capable of scavenging free radical oxygen intermediates. However, due to the very low water solubility and instability of CoQ10 to light, it has low bioavailability [20,85]. Therefore, the construction of a co-delivery system is necessary. Yu et al. constructed chitosan-coated Q10 and Cur co-loaded liposomes, and found that the antioxidant activity of the liposomes achieved a synergistic effect of 41.86 ± 1.84%, which was 5.9 times higher than that of Q10, 2.5 times higher than that of Cur, and 1.7 times higher than that of the mixture. [86]. The results of Zhao et al. [20] showed that multiple lipid particles could improve the loading capacity of lipophilic and hydrophilic active substances with a high level of encapsulation efficiency and long-term stability. The entrapment efficiency of the multilayer structural microparticles was 88.7% for coenzyme Q10 and 77.2% for piperine, respectively. When the piperine and CoQ10 were co-administered, it was found that the bioactivity and oral bioavailability of CoQ10 were enhanced [85]. Likewise, Phycocyanin, a water-soluble pigment in microalgae, displays many bioactivities, such as hepatoprotective, neuroprotective, and antitumor effects. However, it is susceptible to environmental factors such as light, temperature, and pH.

However, the realization of the co-embedding technology is not simple. First, the physicochemical properties of different bioactive substances vary significantly, requiring the wall material to be selected or modified for specific substances during the co-embedding process to optimize the embedding effect. Secondly, how to realize the spatial and temporal control of drug release has become an important challenge in the development of co-embedding technology [87]. In summary, although co-embedding technology shows great potential in improving the performance of different bioactive substances, its complexity and technical difficulties still need further research and breakthroughs.

## 3. Delivery Carrier Classification and Characterization

**Figure 1:**
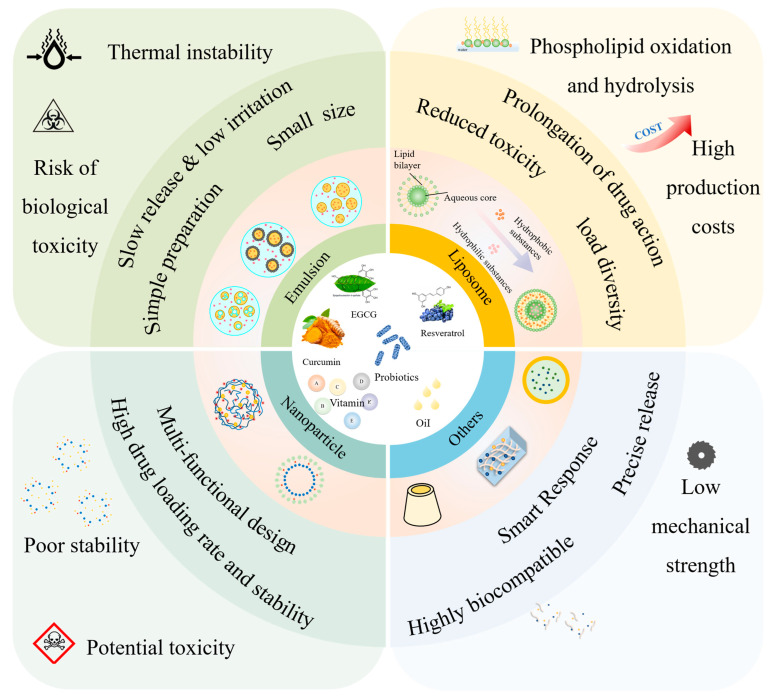
Schematic representation of the classification of co-delivery systems and their functional properties based on carrier type.

In the absence of delivery vehicle encapsulation, synergistic effects may exist between the bioactives themselves, but their effects are limited by factors such as environmental stability, bioavailability, and targeting. Delivery carriers greatly enhance synergistic effects by protecting the active ingredient, modulating the release sequence, and optimizing pharmacokinetics. As shown in Figure 1, the co-delivery system is a carrier that can deliver two or more bioactive substances simultaneously. It has been reported that the overall biological function of multiple-bioactive co-encapsulated substances is higher than the cumulative effect of individual bioactive substances, and the same or better functional effect can be obtained at lower concentrations of bioactive substances [88]. Most delivery systems are limited to encapsulating a single nutrient. Multiple actives can work together to achieve synergistic effects, but simple mixing of actives does not maintain their bioactivity well. Most bioactive substances are characterized by low bioavailability and poor stability, so researchers have used co-delivery systems to improve these shortcomings. Co-delivery systems enable the coexistence of multiple active substances in the same system and can enhance stability, bioactivity, and bioavailability. Co-delivery systems can improve the nutritional value and health benefits of functional food constructed from a variety of bioactive substances. Currently, as shown in Table 2, carriers used for active substance co-delivery systems include emulsions, nanoliposomes, and nanoparticles, among others [89]. Co-encapsulation systems provide a variety of release characteristics to enhance the efficacy and stability of bioactive compounds. These release characteristics include burst release, sustained release, delayed release, triggered release, and directed release. These different mechanisms enhance the functionality of nanocarriers in food applications, meeting the specific needs for controlled and site-specific bioactive delivery [89].

### 3.1. Type of Carrier

Emulsion is a colloidal dispersion system stabilized by surfactants with good interfacial properties, good transparency, and kinetic stability. Encapsulating functional factors in emulsions helps to improve their physicochemical stability and biological efficacy [119]. Nanoparticles are considered promising delivery vehicles for cancer therapy based on their ability to prolong drug circulation time, reduce systemic toxicity, and increase drug accumulation at tumor sites through an enhanced permeation and retention (EPR) effect [120]. Liposomes have several advantages contributing to delivery. They have a role enhancing substance solubility, serving as a sustained release system, providing targeted delivery [121], reducing the toxic effect of substance, providing protection against degradation, enhancing the circulation half-life of active pharmaceutical ingredients (APIs) [11], being effective in overcoming multidrug resistance, improving the therapeutic index of the entrapped substance, and protecting APIs against their surrounding environment [122]. Polymer micelles have been widely used as carriers for the targeted delivery of active substances. Polymer micelles can effectively encapsulate anticancer agents into their hydrophobic core through covalent bonding or physical embedding to assemble them into a nano-sized carrier system. These micelles might extend the retention time of the substance in the blood circulation and preferentially accumulate in tumor tissue through the EPR effect [123].

#### 3.1.1. Emulsions

Traditional emulsion is generally composed of a water phase and an oil phase, which mainly includes single-layer emulsion and double-layer emulsion. Protein-stabilized oil-in-water (O/W) emulsion was an effective vehicle due to the high emulsifying properties and strong ligand-binding capacity of proteins. Fang et al. [81] analyzed that α-tocopherol, resveratrol, and naringenin with different solubilities could be co-encapsulated in one carrier by using the WPI-stabilized oil-in-water (O/W) emulsion. And the results demonstrated that the digestion stability of the active substances could be significantly improved by O/W emulsion. Res improved the digestive stability of α-Tocopherol. α-Tocopherol does not affect the digestive stability of resveratrol or naringenin. Cheng et al. [7] prepared an oil-in-water (O/W) emulsion stabilized by sodium caseinate to achieve the co-encapsulation of the active substance α-Tocopherol with resveratrol. It effectively improved the encapsulation of resveratrol at the oil–water interface and the storage stability of α-Tocopherol and resveratrol, and enhanced the bio-accessibility of α-Tocopherol (~40%) and resveratrol (~90%). Ma et al. found that PTX-VE and 5-FU-TPGS core-matched NEs up-regulated the levels of β-microtubule proteins in the drug-resistant KB-8-5 cell line. The co-loaded emulsions exhibited significant inhibition of tumor growth compared to controls. Some studies had shown that Cur and catechins had synergistic effects in disease prevention and health promotion [124]. The delivery carrier can carry two bioactive substances to increase the nutritional efficacy of Cur and catechin-based nutritional health products. The main difficulty in co-loading two bioactive substance carriers is their different solubility. Cur has high hydrophobicity and is soluble in lipids, while catechins are hydrophilic and insoluble in lipids, which makes carriers suitable for catechin delivery unsuitable for delivering Cur. Therefore, it is very important to develop a co-loading delivery system to encapsulate Cur and catechins, which can protect them from degradation before reaching the site of action. Aditya et al. [9] used the oil-in-water (W/O/W) double emulsion prepared by the two-step emulsification method to embed Cur and catechin. The results showed that the W/O/W double emulsion had a high encapsulation efficiency (88–97%). Compared with free Cur and catechin solutions, the double-layer emulsion could significantly increase the gastrointestinal stability and biological accessibility (four times) of bioactive substances. In addition, the double-layer emulsion co-delivery of Cur and catechin could achieve a synergism of biological activity. The researchers further studied the influencing factors of emulsion core material stability. Cui et al. [52] utilized whey protein isolate fibrils (WPIFs) composed of WPIs and cellulose nanocrystals (CNCs) to form and stabilize W/O/W emulsion loaded with hydrophilic epigallocatechin gallate (EGCG) and hydrophobic Cur. It has good storage stability and physical stability of digestion, and with better resistance to heat and salt compared to the emulsion stabilized by WPIF. It also significantly enhanced the bioavailability of two active substances. Han et al. [92] employed a self-assembled water-in-oil-in-water (W/O/W) double emulsion to encapsulate insulin and quercetin. Through multiple indexes to choose the optimum emulsifier, the black-bean-protein-stabilized W/O/W double emulsion had more stable characteristics. It had higher encapsulation efficiency (insulin: 95.7%, quercetin: 93.4%), lower viscosity, and better emulsifying properties, and this emulsion increased the bio-accessibility of insulin and quercetin by 2.6 and 4.56 times, respectively, while increasing their chemical stability and solubility under a simulated gastrointestinal environment.

Pickering emulsion was a new type of emulsion delivery system that replaced traditional surfactants with solid particles. Colloidal particles adsorbed at the oil–water interface could significantly inhibit the aggregation of droplets by reducing interface energy. The colloidal particles, such as starch, corn protein, pea protein, and whey protein, were widely used to stabilize Pickering emulsions. Liu et al. [49] delivered Cur by using Pickering emulsion stabilized by whey protein isolates (WPIs), lactose (Lac), Maillard-reacted products, and EGCG complexes, which effectively improved the thermal stability of Cur and the percentage retention of Cur in the emulsion. Chen et al. [125] selected two active substances, chlorogenic acid and β-carotene, with different polarities, and used the Pickering emulsion stabilized by shrimp ferritin as the delivery vehicle. Chlorogenic acid molecules were encapsulated in the ferritin cavity while β-carotene molecules were loaded in oil droplets, which could achieve the co-delivery of two active substances and improve their storage stability and centrifugation stability. Guan et al. [126] prepared high internal phase Pickering (HIPE) emulsion stabilized by biosurfactants, lecithin, and silica nanoparticles by a two-step emulsification method. This emulsion could regulate its inner morphology, and lecithin and silica nanoparticles could successfully stabilize this emulsion in a wide pH range. This emulsion was widely used and could be prepared as highly porous poly HIPE monolithic materials with functional surfaces and interconnected porous microspheres. Tang et al. [90] used Pickering double emulsion stabilized by nanoparticles that were prepared by genipin cross-linking pectin–bovine serum albumin for the co-delivery of betanin and curcumin, and the encapsulation efficiency of the two active substances was 65.3% and 84.1%, respectively. The study prepared the emulsion successfully and enhanced the gastrointestinal digestive stability of the active substances, and then increased the bio-accessibility of active substances. The study also showed the synergetic antitumor effect of the two active substances on A549 cancer cells. The particle concentration or proportion of stable Pickering emulsion as a variable also has a great influence on emulsion stability. Niu et al. [37] used chitosan-nanoparticle-based Pickering emulsion to co-encapsulate cinnamaldehyde essential oil (CEO) and chlorogenic acid (CA), and prepared a Pickering emulsion system stabilized by CS-CA nanoparticles with different ratios of CS to CA. The stability of emulsions stabilized by different particles has significant differences. The emulsion stabilized by CS-CA nanoparticles with a ratio of CS to CA of 1:0.75 showed the best stability, and a minimum creaming index value of 26.5 ± 4.6% after 5 days of storage. In addition to the preparation of Pickering emulsion by homogenization and high-speed shear, microfluidization technology is also a good preparation method. Wei et al. [91] discussed the influence of different particle concentrations (0.5–0.3%, *w*/*v*), microfluidization pressures (0–150 MPa), and heating temperatures (30–70 °C) for the physicochemical stability, microstructure, and in vitro digestion of β-carotene-loaded Pickering emulsions stabilized by curcumin-loaded complex nanoparticles. The results found that co-encapsulation has a synergistic effect on improving the photothermal stability of β-carotene and curcumin, and microfluidization at lower pressure promoted lipolysis and enhanced the bio-accessibility of active substances.

In conclusion, the types of emulsion co-delivered actives are mainly focused on O/W emulsions and W/O/W emulsions. Future exploration of W/O emulsions and O/W/O emulsion systems for the co-encapsulation of two or more hydrophilic and hydrophobic bioactive ingredients is needed. Dual-layer emulsions with different compartments are capable of delivering two or more active substances with the same solubility and different solubility at the same time. Moreover, the emulsion encapsulates the active substance to mask the unpleasant taste of the active substance, and thus enables controlled release [127]. However, emulsions can flocculate, agglomerate, and precipitate over time, leading to decreased stability [128]. Therefore, it is necessary to use some molecules to modify the outer layer of the emulsion to stabilize the emulsion.

#### 3.1.2. Nanoparticles

Nanoparticles are usually composed of natural proteins and polysaccharides with diameters ranging from 1 to 1000 nm and have good biocompatibility, subcellular size, and controlled release properties. Currently, nanoparticles can be divided into homogeneous and heterogeneous categories based on the similarities and differences in substances encapsulated by nanoparticles. There are various studies on encapsulating more than two kinds of polyphenols. The study of precision nutrition in the nanoparticle co-encapsulation of various substances mainly focuses on hypoglycemic, anticancer, anti-inflammatory, and antioxidant effects, among others [129,130].

Two insoluble polyphenols were encapsulated by nanoparticles in a system, which could improve the adherence and treatment outcomes of diabetic patients. Novel hyaluronic acid (HA)-functionalized chitosan nanoparticles (CS-NPs) were designed by Hussain et al. [10] for the efficient topical co-delivery of Cur and Res. An ionic-crosslinking technique was used to prepare HA functionalized for co-delivery nanoparticles, which have better colloidal stability. The experimental model of diabetic animals revealed that considerable sustainability was evidenced in nanoparticles after functionalization with HA, and it reduced the local application frequency for managing diabetic wounds, enhanced the therapeutic effect, increased local targeting, and prolonged the residence time of Cur and Res at the target site. A co-delivery nanopolymer was synthesized by Ghobadi-Oghaz et al. [27] using a zein protein as the core and a chitosan polysaccharide as the shell to deliver Cur and berberine (Ber) in MDA-MB-231 breast cancer cells. The results demonstrated a synergistic effect between Cur and Ber on various cancers, and the nanoparticle could successfully increase cell uptake and apoptosis while significantly inhibiting IL-8 pro-inflammatory cytokines. Moreover, the layer-by-layer nanopolymer may be beneficial for use in pharmaceutical products. Zein-propylene glycol alginate-rhamnolipid complex nanoparticles were prepared by Wei et al. [131] using the emulsification–evaporation method to co-deliver resveratrol and the coenzyme Q10. The delivery efficiency and chemical stability of the active substances in the complex nanoparticle were significantly enhanced by rhamnolipid. The sustained release of nutraceuticals from the complex nanoparticle was promoted by rhamnolipid and resveratrol, as indicated by the in vitro simulated gastrointestinal digestion model. Efficient protection and transportation of various active compounds may be provided by the addition of rhamnolipid, allowing their synergistic effects to be exerted. Novel paclitaxel/resveratrol co-loaded albumin nanoparticles (PTX/RES NPs) were developed by Zhao et al. [11] to achieve synergistic anticancer efficacy and overcome the drug resistance of PTX. PTX/RES nanoparticles could be efficiently internalized by cells, and also showed dramatic in vitro cytotoxicity against naturally occurring cancer cells and MDR cancer cells, and exerted a synergistic combination efficacy of the two drugs. The in vivo antitumor experiments demonstrated that the antitumor effect of the hybrid nanoparticle was better than the single drug-loaded nanoparticle or a free drug combination. Curcumin-based anticancer nanoparticles were prepared by Jaiswal et al. [96] using the anti-inflammatory enzyme serratiopeptidase through the desolvation method. The stability and anticancer activity of Cur were efficiently enhanced by the prepared nanoparticles, which also effectively exerted tumor inhibition effects and demonstrated anticancer activity in MCF-7 and HeLa cell lines. Multilayer structural nanoparticles (MSNPs) were prepared by Wang et al. [64] using gliadin (Gli), carboxymethyl konjac glucomannan (CMK), and chitosan hydrochloride (CHC) to co-encapsulate fucoxanthin (FUC) and Cur. Lyophilized nanoparticles exhibited excellent water redispersibility after the addition of CHC. In addition, the CHC coating significantly improved the physical stability, storage stability, and thermal stability of MSNP. The nanoparticle was shown to be able to effectively delay the photo-degradation and thermal degradation of the encapsulated FUC and Cur. Moreover, the prepared nanoparticles exhibited programmed sequential release properties, which enabled the delivery of Cur and FUC in the small intestine and colon. Endothelial cell-targeting and reactive oxygen species (ROS)-ultrasensitive nanoparticles were exploited by Hou et al. [26] to mediate efficient co-delivery of VCAM-1 siRNA (siVCAM-1). The nanoparticle significantly mitigated neutrophil infiltration into ischemic myocardium and provoked potent anti-inflammatory efficacy. SiVCAM-1 and DXM synergistically inhibited neutrophil recruitment to damaged myocardium, reduce myocardial inflammation, and promote myocardial recovery in MIRI rats.

Additionally, other researchers further investigated the influence of nanoparticles encapsulating polyphenols of different polarities on their structural properties and functional activity. The influence of the interaction between protein and bioactive compounds was studied by Dong et al. [98]. Nanoparticles formed by the interaction between bovine serum albumin, α-tocopherol, resveratrol, and EGCG were observed to decrease the masking effect on the antioxidant activity of these compounds and reduce the antagonism among them. The structural integrity and antioxidant properties of α-tocopherol, resveratrol, and EGCG were maintained by these tri-ligand complexes. Multiple targeting and layer-by-layer release could be achieved by the nanoparticles through modification with different characteristic modifiers due to their layered structure. A dual cancer-targeted nanoparticle system was applied by Chu et al. [97] to co-deliver EGCG and CU. The nanoparticles consisted of hyaluronic acid, fucoidan, and polyethylene glycol gelatin. In addition, a dual-targeting system of hyaluronic acid and fucoidan was established for targeting CD44 on prostate cancer cells and P-selectin in tumor blood vessels, respectively. In mice, cancer-targeted EGCG/CU-loaded nanoparticles significantly reduced orthotopic tumor growth without causing organ damage. In conclusion, the dual-targeted nanoparticle co-delivery system of EGCG and CU greatly enhances its synergistic effect in cancer therapy, suggesting that it has great potential for developing therapeutic approaches for prostate cancer.

At present, some novel nanoparticle preparation methods have also been developed to effectively improve the stability of nanoparticles and the bioactivity of active substances. Nanoparticles were prepared by Feng et al. [21] adopting a multiple interaction-based strategy via a two-step flash nanoprecipitation (FNP) process to co-deliver Cur and procyanidins (PC) with different polarities. The core–shell structure of the NP greatly enhanced the thermal–light stability of the encapsulated PCs and Cur. A synergistic antioxidation effect was found between PC and Cur, and the nanoparticle further improved the antioxidation efficiency, demonstrating better encapsulation performance, with the embedding rate being higher than 95%, and the load rate was higher than 38%. In vitro release experiments showed that the nanoparticle has favorable pH responsiveness in the vast gastrointestinal pH gradient, effectively preventing the leakage of wrapped bioactive substances in the stomach pH environment, which benefits the intestinal delivery of the bioactive substances. Hydrophilic egg white-derived peptide (EWDP) and hydrophobic Cur self-assembled amphiphilic nanoparticles were prepared by Yang et al. [132] based on carboxymethyl chitosan (CMCS) shells and γ-cyclodextrin (γ-CD) cores. Due to the introduction of an additional hydrogen bonding network and hydrophobicity, EWDP could synergistically produce excellent colloidal properties with CMCS and improve the water solubility of Cur. Moreover, the overall antioxidant activity, bio-accessibility, gastrointestinal stability, and Caco-2 cellular uptake were significantly improved in the presence of EWDP. All these validate that nanoparticles can enhance the stability and bioactivity of co-administered actives. Similarly, novel core–shell-type surface-engineered nanoparticles were prepared by Chen et al. [133] to co-encapsulate coenzyme Q10 and piperine, which exhibited better physicochemical stability and thus extended the shelf life of their transformed commercial products. The smaller size of nanoparticles was beneficial to enhance the absorption of intestinal epithelial cells [134]. In addition, nanoparticles smaller than 500 nm are not easy for macrophages to remove, which is conducive to improving the cycle time of bioactive substances. Nanoparticles easily cross biological barriers and are deposited in organs for easy absorption by the body [135]. In addition, co-delivery has a synergistic effect that can improve tumor inhibition efficiency through several different targets and has the potential to reduce side effects and maximize drug efficacy [136]. By co-delivering multiple active substances, it is also possible to reduce the problem of drug resistance caused by single-drug use.

#### 3.1.3. Liposomes

Compared with other delivery systems, liposome-delivery systems have additional advantages, such as biocompatibility, non-immunogenicity, self-assembly ability, and the capability to load both hydrophilic and hydrophobic substances while improving their solubility. They also possess the ability to achieve large payloads, protect encapsulated substances from external influences, reduce the toxicity of encapsulated substances, and minimize the exposure of sensitive tissues to toxic drugs [137]. Furthermore, liposome-delivery systems enable site-specific targeting and enhance tissue penetration [138]. Liposomes can encapsulate hydrophilic compounds in their core and incorporate hydrophobic molecules into their bilayers. Nanoliposomes, composed of phospholipids and cholesterol, are the main components of biofilms and have biological advantages such as biodegradability, biocompatibility, and extremely low toxicity. Nanoliposomes consist of a hydrophilic core and one or more lipid bilayers, which can encapsulate hydrophilic components into their core cavity or dissolve hydrophobic components into their phospholipid bilayers. Novel liposomes were focused on by Xu et al. [60] to co-deliver hydrophobic docosahexaenoic acid (DHA) and hydrophilic anthocyanidin, and their vesicles exhibited uniform size distribution and spherical shape. The novel docosahexaenoic acid (DHA)–anthocyanidin-co-delivery liposome (DA-LP) bilayer membrane remained intact in Simulated Gastric Fluid (SGF), while liposomal phospholipids were hydrolyzed and remarkably released nutrients during simulated infant intestinal digestion. Furthermore, DA-LPs had no cytotoxicity to Caco-2 cells and significantly increased cell uptake of DHA and anthocyanidin. This study would be valuable for the development of new biphasic/complex liposomes or other delivery systems for application in infant foods. Nanoliposomes loaded with copper peptide, acetyl tetrapeptide-3, and myristoyl pentapeptide-4 (CAM-NLPs) were developed by Tian et al. [139] to simultaneously achieve a synergistic effect of multiple bioactive peptide combinations and improve their delivery efficiency for promoting hair growth. The final prepared nanoliposomes exhibited uniform particle sizes, high encapsulation efficiency, and loading capacity for the bioactive peptides. In vitro experiments demonstrated that these nanoliposomes are an effective transdermal co-delivery nanocarrier for alleviating hair loss in androgenetic alopecia with hair growth promotion, high safety, and great potential.

Modification of the outer layer of conventional liposomes prolongs the circulation time of the drug in vivo, improves stability, and ensures the stability of the encapsulated substance during storage [140]. Modification of chitosan on the outer layer of conventional liposomes effectively improved the stability of conventional liposomes, which exhibited higher antioxidant activity than the mixture and liposomes alone in 10 weeks of storage due to the effective inhibition of liposome swelling and the release of active substances by the chitosan coating [5]. The surfactant rhamnolipid was used by Ji et al. [141] to modify conventional liposomes, utilizing β-carotene (βC) and rutinoside (Rts) to generate co-encapsulated liposomes by ethanol injection. The RL-complex liposomes loaded with βC and Rts (RL–βC–Rts) showed higher loading efficiency and good physicochemical properties (size = 167.48 nm, zeta-potential = −5.71 mV, and polydispersity index = 0.23). Compared with other samples, the RL–βC–Rts showed better antioxidant activities, antibacterial ability, and storage stability. In in vitro digestion experiments, βC exhibited good release kinetic properties. Cinnamon extract (CE) and zein hydrolysate (ZH) were co-encapsulated with nanoliposomes (NLPs) by Imani et al. [142]. The study found that the synergistic antioxidative relation between CE and ZH provided a high antioxidant activity that caused a reduction in lipid oxidation in the NLPs, and NLPs co-loaded with CE and ZH with the lowest deformation and smallest particles. A strategy of a liposome co-delivery system for stilbene (trans-resveratrol) and flavanone (naringenin) was developed by Huang et al. [93], which demonstrated desirable encapsulation efficiency and improved environmental stability. Liposome co-encapsulation of trans-resveratrol and naringenin also increased the antioxidant activity of scavenging DPPH radical ability, inhibiting lipid peroxidation capacity and reducing power compared to single polyphenol-loaded liposomes. A potential combined therapeutic strategy for modulating the tumor immune microenvironment and glioma-targeted drug delivery system was developed by Zheng et al. [143]. The combination therapy was achieved through remodeling tumor metabolism and tumor immune microenvironment by modulation of the mTOR (mammalian target of rapamycin) pathway. A liposome remotely loaded with shikonin (a potent ICD stimulus) was developed by Li et al. [94], which demonstrated the ability to effectively induce ICD at high dosage in vivo. The dual-loaded liposomes were designed to load the effective ratio, which was better internalized by cells compared to physical mixtures.

The bioavailability and stability of active substances differ due to the different preparation methods of nanoliposomes. Lecithin-based nanoliposomes were utilized by Khatib et al. [95] to co-encapsulate lupulon and xanthohumol as major bioactive components of hop using the sonication method. The optimized nanoliposomes had high antioxidant activity equivalent to xanthohumol and high antimicrobial activity equivalent to lupulon. The preparation method of liposomes also has a great influence on its properties. The noisome was screened by Joshi et al. [144] based on its particle characteristics and encapsulation efficiency, which was produced using a microfluidics system. The result showed that the particle characteristics and encapsulation efficiency of liposomes not only depend on microfluidic process parameters but also on the properties of the surfactant used to produce the niosome carriers. Tween 60 niosomal containing antioxidant molecules for the encapsulation of oxidative stress, gallic acid, ascorbic acid, curcumin, and quercetin was developed by Tavano et al. [145]. The aim was to improve the nutritional quality of dairy products and to make them useful in the prevention of many diseases caused by oxidative stress. The result verified that their physicochemical property and the encapsulation efficiency relative to the preparations containing a single antioxidant were influenced by the co-encapsulation of the mixture of gallic acid/curcumin and ascorbic acid/quercetin. In addition, the release of antioxidants appeared to improve, and their combination resulted in a promoted ability to reduce free radicals because of a synergistic antioxidant action.

#### 3.1.4. Other Delivery Carriers

Many active substances can be co-delivered by a variety of carriers, except the above co-delivery carriers, including gels (hydrogels, oil gels, emulsion gels), micelles, cyclodextrins, and new delivery carriers such as ferritin cages. Emulsion gels are structured emulsion systems which have emulsified oil droplets captured within three-dimensional networks formed by cross-linking polymers (including proteins and polysaccharides). Emulsion gels are good delivery systems for multiple bioactive compounds, which can protect them from degradation and control their release during digestion. Emulsion gels are used as fat substitutes to imitate the textural and rheological characteristics of fat, and even to adjust the volatile release in fat-reduced food. Chen et al. [146] developed alginate-based emulsion gels that were composed of different oil fractions (0–20%), and interfacial compositions were used to simultaneously encapsulate lipophilic and hydrophilic bioactive substances. Emulsion gels were able to protect both lipophilic and hydrophilic bioactive substances, and those substances encapsulated with oil droplets could be more retained during heating. Furthermore, the simultaneous encapsulation of the two bioactive substances could produce a synergistic effect to improve their chemical stability, which would be beneficial for the development of functional food with multiple bioactive substances exploited.

Moreover, oleogels are known as the future fats, and their main application is in replacing harmful saturated and trans fatty acids with the structure of healthy unsaturated oils or fatty acid-rich oils. Meanwhile, oleogel is explored as a vehicle to enhance the bioavailability and bio-accessibility of hydrophobic/lipophilic functional components because it can be a hydrophobic medium and provide a unique structure. Emulsion-templated medium-chain triglyceride (MCT) oleogel was prepared by Kavimughil et al. [65] as a co-delivery vehicle for curcumin and resveratrol. The oleogel showed higher bioavailability, and the total bioaccessible fraction increased up to 1.13 and 1.2 for Cur and Res, respectively, compared to the control MCT oil. Similarly, the research found that the MCT oleogel significantly enhanced the permeation of Cur and Res more than the control MCT oil. The encapsulation and protection of the bioactive compounds could be achieved by emulsions, using additive layering to achieve targeted controlled release of active substances. In addition, hydrogels are water-soluble polysaccharides and are widely used in food, non-food, pharmaceutical, and medical applications as thickeners, emulsifiers, viscosifiers, and gelling agents. A pH-sensitive double-emulsion-filled hydrogel simultaneously loaded with astaxanthin and phycocyanin was prepared by Yu et al. [147] using the two-step emulsification process. The maximum encapsulation efficiency of astaxanthin and phycocyanin was 94.1% and 90.82%, respectively. In vitro digestion proved that the double-emulsion-filled gellan gum hydrogel could be used as a pH-sensitive carrier and for the intestinal targeted delivery of hydrophobic and hydrophilic food bioactive ingredients. The crystalline and well-organized network structure of iota-carrageenan fibers was utilized by Yao et al. [109] to encapsulate Cur and Res. The effect of encapsulation time on hydrogel has been studied. The result showed that Res was better suited to entrap in the iota-carrageenan network, which had better storage stability. The two nutraceuticals are easily protected from heat by the IC network and released in a sustained manner. Bigels of oleogel-in-hydrogel structures were developed by Lu et al. [56] as vehicles for curcumin and epigallocatechin gallate (EGCG), and the structural characteristics of bigels were significantly affected by the content of the oleogelator glycerol monostearate. The result showed that the structures of bigels could be well adjusted by the oleogelator glycerol monostearate, which then affected the release of the functional ingredients encapsulated, and were beneficial for the development of functional food. The drug was encapsulated in a hydrophobic core by polymer micelles and effectively delivered to the treatment site, which could enhance the bioavailability of the drug and reduce side effects. A co-delivery system of doxorubicin and PTX based on a folate-conjugated and pH-sensitive polymeric micellar system was carefully designed and synthesized by Niu et al. [113], and the results verified that the polymeric micelles were internalized in the cytoplasm via endocytosis, and the cellular uptake was enhanced by folate modification. The co-delivery of DOX and PTX has ideal anticancer efficiency in vitro that because of the active targeting, the synergistic effect of drug co-delivery, and the folate modification of polymer micelles. Chitosan-coated hyaluronic acid micelles (R/C/D@HAssOA) were developed by Song et al. [114] to co-deliver doxorubicin (DOX) and the programmed-death ligand, which enhanced the antitumor effect through the combination of active substances. The study showed that the antitumor effect of DOX was significantly enhanced by the co-delivery system, which reduced the cardiotoxicity of DOX. A pH-sensitive polymer, poly (ethylene glycol)-benzoic imine-poly(γ-benzyl-l-aspartate)-b-poly(1-vinylimidazole) block copolymer (PPBV), was synthesized by Yang et al. [148]. Subsequently, a pH multistage responsive micellar system was developed for co-delivering PTX and Cur, which synergistically eliminated breast cancer stem cells (bCSCs) and non-bCSCs. This pH multistage responsive micellar system could reduce its size after long circulation periods at tumor sites and extravasation from leaky blood vessels, thus facilitating its cellular uptake and deep tumor penetration.

The most widely used water-soluble carriers are cyclodextrins, which have found many applications in pharmaceutics, agriculture, and chemistry science and the most, by far, in food science. The cavity diameter of β-cyclodextrins is well-suited for encapsulating molecules of the size of phenols. For this reason, β-cyclodextrin is most used as an encapsulating agent. The aqueous solubility of natural cyclodextrins is very poor; nevertheless, it can be significantly improved by chemical substitutions at the 2, 3, and 6 hydroxyl sites. The complexes formed by two polyphenols, trans-Ferulic acid (FA) and Gallic acid (GA), with 2-hydroxypropyl-β-cyclodextrin (HPβCD), were studied by the spray-drying method by Olga et al. [112]. The studies indicated that the simultaneous encapsulation of the two polyphenols had no significant differences in the antioxidant efficiency, and the co-encapsulated compound of the two polyphenols exhibited a possible interaction in the cyclodextrin cavity. In recent years, researchers have discovered a novel delivery vehicle: gel-like plant ferritin. Ferritin is an iron storage and transport protein widely found in living organisms and consists of 24 subunits self-assembled into an ordered nanocage structure. Its cage structure can encapsulate small molecule cargoes, and its shell is enriched with a variety of active amino acid residues, which facilitate the conjugation of functional components. The reversible self-assembly property of ferritin makes it easy to encapsulate active substances, and the nano-size facilitates the enhancement of permeability and retention effect, which can be used for active and passive tumor targeting with low immunogenicity and good biocompatibility. In addition, ferritin can be prepared at low cost and high yield by the E. coli expression system. The hydrophilic EGCG and the hydrophobic quercetin were simultaneously enriched in the cavity of red bean seed deprived of iron (apoRBF) phytoferritin by Meng et al. [149], and the stability of the two active substances was enhanced. The stability and bioavailability of delivery active substances have been enhanced by modifying the outer layer of phytoferritin. The red bean seed ferritin (RBF)-epigallocatechin (EGC)-chitosan nanoparticle (REC) was prepared by Yang et al. [150] using a simple one-step method. EGCG could be encapsulated in phytoferritin without acid–base transfer. The size of REC nanoparticles when heated is 12 nm. REC was particularly suitable for the encapsulation and stabilization of pH-sensitive molecules, which could improve the stability of ferritin against enzymatic digestion and increase the retention of encapsulated food compounds such as polyphenols.

Current research on co-encapsulation systems focuses on the precise design and functional optimization of multi-component co-delivery systems. Co-encapsulation systems for the delivery of bioactive ingredients need to be rationally designed to balance the loading capacity, stability, and release characteristics for active substances with different physicochemical properties [4]. The study points out that the chemical compatibility between the carrier and the embedded substance and the ratio between the substances are the key to determining the synergistic effect, and that smart-responsive materials are needed to realize the spatially and temporally controllable sequential release [151]. Recent advances emphasize the integration of targeting ligands and stimulus response modules into the delivery system to achieve “triple release” (timing, dosing, and localization) through dual physicochemical–biological regulation, which needs to be elucidated by combining molecular dynamics simulations and in vivo and ex vivo delivery trajectory tracking techniques to elucidate the carrier–activity interaction mechanism [152]. Future breakthroughs lie in the development of multimodal approaches and the development of a multimodal characterization platform to establish the quantitative relationship between the structural parameters of the drug-carrying system and pharmacokinetics to provide theoretical support for precise nutritional interventions. In summary, emulsion carriers are dominated by the droplet structure formed at the interface of the oil and water phases, which is suitable for the encapsulation of lipid-soluble substances. The micron-sized particle size results in limited biological barrier penetration, and functional emulsifiers need to be developed to enhance drug loading and targeting. The phospholipid bilayer structure of liposomes allows them to encapsulate both water-soluble and lipid-soluble substances. In addition, they can be modified on the surface to extend the circulation time or coupled with targeting ligands to enhance targeting. However, there are drawbacks such as drug leakage and lack of stability, and environment-responsive liposomes need to be investigated to realize precise release. Nanoparticles can be loaded with multiple substances at the same time, but unmodified nanoparticles may suffer from poor stability. Existing carriers are difficult to adapt to the differences in physicochemical properties (e.g., polarity, release kinetics) of different actives, and the preparation process of co-delivery carriers is not yet perfect, which poses a challenge for large-scale production and quality control. Therefore, the development of co-delivery carriers that can respond to specific physiological environments to realize intelligent drug release, improve therapeutic efficacy, and reduce side effects is a possible future research direction. Liposomes, nanoparticles, and emulsions have developed a more mature technological system for the co-loaded delivery of polyphenolic active ingredients. Liposomes can encapsulate lipid-soluble substances through a bilayer phospholipid structure, which can significantly enhance their water solubility and gastrointestinal stability. Emulsions are widely used in the sustained-release delivery of fat-soluble vitamins (e.g., vitamins A, D, and E), where controlled release is achieved through stabilization at the oil–water interface. However, water-soluble vitamins (e.g., vitamins C and B) and polyphenol–vitamin complex systems have been relatively poorly studied, mainly due to the difference in compatibility between their hydrophilicity and emulsion structure [153,154]. Current nutrient co-delivery systems focus on the steady-state release of nutrients, such as smart carriers for controlled-release co-delivery of active substances, but their clinical translation is still limited by a lack of validation in human trials. In the future, personalized delivery solutions need to be developed by combining multi-omics data (metabolomics, microbiomics). In addition, breakthroughs in mucosal barrier penetration and cell-selective delivery are needed to achieve precise intervention in metabolic diseases such as obesity and diabetes.

### 3.2. Methods for Characterization of Co-Delivery Structures

**Figure 2:**
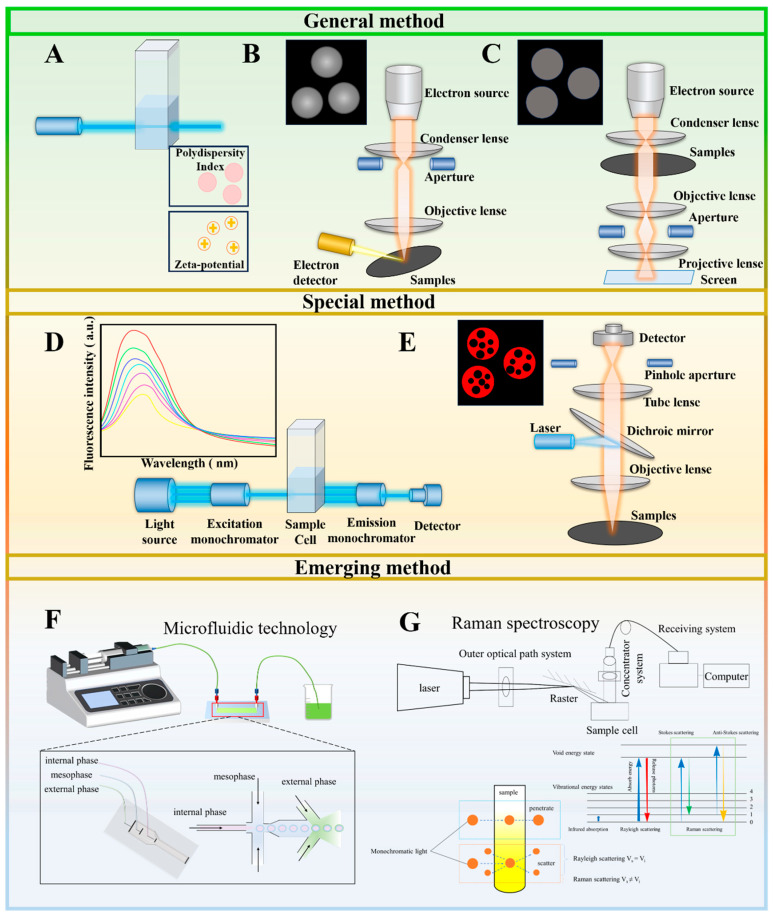
Schematic diagram of the co-delivery system characterization technique. (**A**) Dynamic light scattering; (**B**) scanning electron microscopy; (**C**) transmission electron microscopy; (**D**) fluorescence spectrum; (**E**) confocal laser microscopy; (**F**) microfluidic technology; (**G**) Raman spectroscopy.

In the study of co-delivery systems, numerous methods are used to characterize the structure of the delivery system (Figure 2). Structural characterization methods are classified according to their applicability in conventional general-purpose methods and dedicated methods. Conventional general-purpose methods can be widely applied to most co-delivery carriers and mainly include the characterization of the physical structure of the carrier (dynamic light scattering), microstructural observation (transmission electron microscopy and scanning electron microscopy), and interaction analysis (labeling techniques with fluorophores). Proprietary methods can characterize the structural properties of specific carriers more accurately and with a high degree of specificity than general methods, which have a narrower range of applications, including mainly confocal microscopy techniques and fluorescence spectroscopy.

#### 3.2.1. Conventional Generic Method

Currently, there are numerous studies on the encapsulation of active substances using co-delivery carriers, which are often applied to functional foods or disease treatment, so what cannot be ignored is their bioavailability in the human body. To ultimately achieve high bioavailability, it was inevitable that all the properties of the carriers needed to be optimized during the preparation process. In addition, whether the active substance was successfully encapsulated or not also needed to be measured and compared. Most of the delivery carriers needed to be digested in the human body and eventually reach the small intestine, and finally be absorbed and utilized by the small intestinal epithelial cells to maximize their bioactivity. The particle size will affect the delivery effect of the carriers when the co-delivery carriers are often required to encapsulate two or more actives. Dynamic light scattering (DLS) is probably the most commonly used optical technique for sizing nanoparticles dispersed in fluid [155]. In detail, it focuses on the diffusion coefficients of the particles that can be obtained by receiving the light scattered by the particles, then calculating the autocorrelation function of the intensity of the scattered light, and finally determining the size of the particles based on the Stokes–Einstein function [156]. DLS is routinely utilized in many laboratories worldwide, with applications ranging from industrial production control to the fundamental study of interacting particle systems [157]. Particle size, potential, and PDIs are the main evaluation indicators of DLS, which are generally used to assess the stability of co-delivery carriers (emulsions, nanoliposomes, and nanoparticles). The delivery system had a narrow and uniform particle size distribution, with PDI < 0.3 and high ζ-potential value (±30 mV or higher), which indicated that the delivery carrier had higher stability and was conducive to the co-delivery of bioactive substances [158,159]. The stability of the water-in-oil-in-water double emulsion that co-delivered hydrophobic curcumin and hydrophilic catechin is preliminarily judged by the results of DLS [9]. The carriers remained small and stable after co-loading the two actives, and the smaller increase in the particle size could also corroborate that the actives are successfully encapsulated into the nanoparticles [5,101].

In addition to the study of the co-delivery vehicle concerning DLS, the study of the microstructure was important. The observation of the image of the co-delivery vehicle directly showed its actual shape, the interaction between the active substance and the vehicle, and the distribution of the active substance. What is more, it also revealed whether the active substance was well encapsulated or not. Transmission electron microscopy (TEM) is a powerful tool used to characterize morphology, crystal structure, and chemical composition at an atomic resolution [160]. Another advanced microscopy approach, TEM, provides detailed information concerning the internal structure of nano/micro-carriers, shape heterogeneity, and aggregation tendency [161]. It provides local information about the surface and bulk of samples at the atomic scale and also reveals chemical, electronic, and three-dimensional structural information [162]. TEM observation was often used to understand the morphology of co-delivery carriers and to compare the changes before and after the encapsulation of active substances, and particle size and PDI can also be visualized through TEM images. Chen et al. found that the majority of the prepared liposomes are typically round with no obvious aggregation by TEM [24]. The comparison of TEM images between different samples allows for a preliminary judgment on the success of the modification of the outer layer of the co-delivery carrier at the structural level. Amjadi et al. [163] prepared inulin-coated nanoliposomes, and the spherical structure of the liposome vesicles could be seen in the TEM images. TEM images of inulin-coated samples show a spherical nucleus–shell structure, with a distinctive lamina around the vesicles. This phenomenon confirms the success of the surface coating of NLPs with cationic inulin. Yang et al. [101] found that the nanoparticles display a typical nanoscale structure with a particle size of 150–200 nm by TEM images, which is consistent with the DLS results. Gao et al. [164] also observed by TEM that liposomes co-loaded with amphiphilic and curcumin prepared by microfluidic control showed a nanoscale spherical shape. Compared to blank nanoparticles, TEM images reveal that the nanoparticles after the co-encapsulation of the two actives presented a more ordered and compact spherical structure. Likewise, scanning electron microscopy (SEM) is widely used in various fields such as medicine, biology, biotechnology, and industry. This method provides the possibility of examining surfaces and substrata, as well as chemical analysis in micro- and nano-dimensional materials. SEM utilizes electrons for imaging samples to facilitate the visualization of microstructures [165]. The studies by SEM are generally divided into microscopic and spectroscopic groups, where the former is used for evaluating phases, particles, and morphology [166]. Compared with other electron microscopy technologies, SEM is characterized by its rapidity, large scanning range, and facility of operation [167]. SEM samples are usually solid, and the three-dimensional structure of the co-delivery carrier can be observed, as can the surface loading morphology and depression cracks. Menegazzi et al. [74] find that the spray-dried microcapsules have a spherical shape, sleek surface, and slight depressions. SEM is often used to assess the stability of carriers at the structural level. The co-delivered core–shell microspheres prepared by the electrospraying technique have a uniform and smooth spherical morphology and exhibit a controlled size distribution [168,169].

DLS eliminates the need for complex sample pre-treatment and allows for rapid measurement of the hydrodynamic diameter and particle size distribution of nanoparticles in solution. The true state of the carrier under physiological conditions (e.g., aggregation or dispersion) can be preserved. This requires a low sample volume and is suitable for real-time stability monitoring [170,171]. However, DLS has limited resolution and cannot observe carrier morphology, internal structure, or distribution [172]. TEM has ultra-high resolution (sub-nanometer level), which can observe the carrier morphology, core–shell structure, internal pores, and drug distribution [173,174]. Particle size distribution can be accurately calculated by image statistics, avoiding the polydispersity error of DLS. However, samples need to be dried, ultra-thin sectioned, or negatively stained, which may introduce structural artifacts (e.g., collapse or deformation). Only a very small area can be characterized, and multiple areas need to be sampled to ensure representativeness. SEM provides three-dimensional surface structure information and is suitable for observing carrier roughness, porosity, and surface modification [175]. Compared with TEM, it is easier to obtain the overall morphology of micrometer-scale structures. However, it is difficult to analyze the fine internal structure. Non-conductive samples need to be sprayed with gold or plated with carbon, which may hide the surface details [176]. Only the surface can be imaged, and the internal drug loading cannot be directly observed. Therefore, the three techniques are often used in conjunction to fully characterize co-delivery carriers.

#### 3.2.2. Specialized Methodology

Some carriers have structural particularity, and it is difficult to elucidate their principle and mechanism by conventional characterization methods, so scholars have developed special methods to characterize these co-delivery carriers. The confocal microscopy technique, as a powerful approach for imaging delivery vehicles, has been utilized to attain 3D reconstructions and high-resolution micrographs via a spatial filter eliminating beam aberrations. The capability of CLSM in the non-invasive visualization of a broad range of micro- or nano-colloidal, solid, and even liquid systems offers valuable possibilities to accurately characterize their physical attributes in vitro, and investigate the biological performance of bioactive/drug-comprising carriers in situ [161]. Confocal fluorescence microscopy was utilized to analyze the interfacial structure by labeling nanoparticles and the oil phase. The successful preparation of the co-delivery carrier can also be predicted by the distribution of the oil phase and the water phase. CLSM is also the most common method of visualizing the internal structure of emulsions [125]. Among co-delivery carriers, CLSM is mainly applied in carriers containing both water and oil phases. Nile red was used to label the oils in the emulsion samples, and observation of the samples through CLSM revealed that the oil droplets contain numerous internal droplets, which are distributed in a relatively homogeneous manner. This indicates the successful preparation of the W/O/W double emulsions [92]. A nano-container system based on Fe²⁺ ion coordination self-assembly has been developed by Lin et al. [177] for the simultaneous delivery of the chemotherapeutic drug doxorubicin and miRNA. CLSM was used to observe the intracellular localization of nanoparticles, lysosomal escape, and drug release processes. The synergistic delivery effect of both was confirmed by CLSM dual-channel imaging. Fluorescence spectrum could be used to detect the polarity of the surrounding environment of the intrinsic fluorophore of proteins, which was consistent with the binding between proteins and other compounds [178]. Fluorescence spectroscopy is a useful means of characterizing changes in the conformation and interactions of proteins and other fluorescent substances in nanoparticles; thus, fluorescence spectroscopy is often used as a co-delivery carrier for protein interactions. After the co-encapsulation of resveratrol and CoQ10 into complexed nanoparticles, it was demonstrated by fluorescence spectroscopy that zein, PGA, resveratrol, and CoQ10 were complexed through intermolecular interactions, leading to an altered microenvironment of hydrophobic residues. In a study by Yang et al. [179], a decrease in the emission intensity of resveratrol was observed after its encapsulation by nanoparticles, which suggested that hydrophobic interactions and hydrogen bonding were formed between zein and resveratrol molecules within the nanoparticles.

While rich and diverse, current methods for characterizing delivery systems have limitations in monitoring multiple active substances interacting with carriers in complex systems in situ and in real time. CLSM requires fluorescent labeling of the drug or carrier, which may alter its physicochemical properties, such as surface charge and stability, and is ineffective for imaging deep tissues or highly scattered samples. Fluorescence spectroscopy, on the other hand, may mask the signal due to background fluorescence or scattered light, and steady-state fluorescence spectroscopy struggles to capture fast dynamic events. In addition, the dynamic process of active substance release from carriers in physiological environments is difficult to track accurately without disturbing the system. Meanwhile, some characterization techniques require expensive equipment and specialized operators, limiting their wide application. Emerging technologies such as microfluidics can be used for rapid detection of size distribution and encapsulation rate of liposomes or nanoparticles, avoiding the limitations of traditional DLS for polydisperse systems. Surface-enhanced Raman spectroscopy can be used to dynamically monitor drug release behavior and local microenvironmental changes within cells. Single-particle tracking techniques can be used to analyze the penetration efficiency of targeted ligand-modified nanoparticles in the tumor vasculature. With the rise of intelligent delivery systems (e.g., optical, magnetic), characterization techniques are moving toward real-time, in situ, and high spatiotemporal resolution.

### 3.3. Factors Affecting the Release Kinetics of Co-Delivery Carriers

#### 3.3.1. Different Digestion Models

To develop co-delivery vehicles for the delivery of bioactive compounds, an understanding of the physiological and physicochemical environment of the human digestive system is unavoidable. In vitro digestion models are known to be effective in predicting the oral bioavailability of nutrients. However, most of the in vitro digestion studies so far have been conducted using static digestion models because of their simplicity and the cheap and easy availability of experimental materials [180]. However, due to the simplification of the digestion process, it is impossible to simulate the mechanical forces, fluid dynamics, and changes in the biochemical environment during digestion. In contrast, the TNO gastrointestinal model (TIM-1), which is a computer-controlled dynamic digestion model, takes into account experimental factors such as peristaltic movements, gastrointestinal residence time, and elimination of non-absorbable samples. Based on this, this paper compares different digestion models [181]. Lu et al.’s [180] study on TIM-1 and pH static digestion revealed the effect of milled starch granules on stabilized Pickering emulsion on curcumin bio-accessibility. Simulations in the static model showed that the bioavailability of curcumin encapsulated in Pickering emulsion was 27.6%. In contrast, the bioavailability of curcumin in the emulsion system was 50.7% when using the TIM-1 model. The TIM-1 model elucidated well the mechanism of digestion of Pickering emulsion stabilized by ground starch granules in the upper gastrointestinal tract. The gradual release and improved dissolution characteristics of the milled starch granule-stabilized Pickering emulsion highlight its potential as a delivery system for lipophilic bioactive compounds. The same findings were found in Li et al. [182]. In contrast to the static digestion model, higher starch hydrolysis was observed in the TIM-1 model during the gastric and intestinal digestion phases (*p* < 0.05). The TIM-1 model is considered a closer estimate of the functional effect of cinnamon on starch hydrolysis than the static model. Although dynamic in vitro digestion models (e.g., TIM-1) have been widely used to assess the bioavailability of single dietary components [183], their application in food co-delivery systems is still in the exploratory stage; especially, the systematic comparison of dynamic and static digestion models in the study of co-delivery mechanisms is still relatively lacking. Most of the current co-delivery studies still rely on static models, and their fixed experimental conditions may ignore the effects of key dynamic factors, such as mechanical shear and phase emptying, on the synergistic release of co-delivered components [184]. Therefore, future studies need to systematically compare the in vitro release data of TIM-1 with those of the static model, focusing on the spatial and temporal interactions (e.g., interfacial stability, sequence of component release, and synergistic effects) between the delivery vehicle and the digestive environment under dynamic conditions. Such comparisons will not only elucidate the regulation of co-delivery efficiency by dynamic factors, but also provide empirical evidence to optimize the carrier design, thus promoting the development of personalized nutritional formulations.

#### 3.3.2. Release Dynamics Modeling

**Figure 3:**
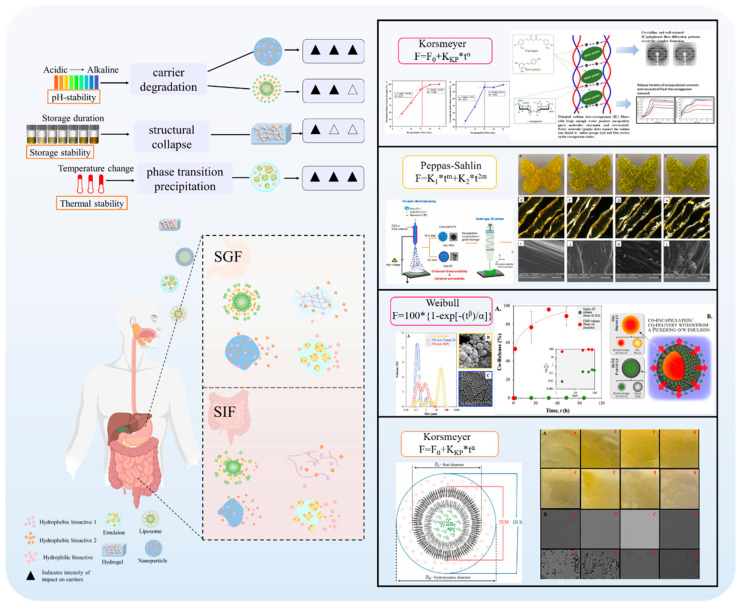
Schematic representation of the ex vivo stability and release kinetics of the co-delivery vehicle, data reproduced from [109]. Photographs in the Peppas-Sahlin model (**A**–**D**) are 3D printed gelatin hydrogels; optical microscopy images (reflections; scale 1 μm) (**E**–**H**); and SEM images of nanoparticles at 5000× magnification (**I**–**L**), data reproduced from [108]. Weibull model in which A (**left**) represents the sample particle size distribution, B and C (**right**) represent SEM images. A (**right**) is the co-release profile of oil-in-water droplets and the lipid particles stabilizing them, respectively. B (**right**) Schematic illustration of the separation of the co-encapsulation of the actives in the microstructure of the o/w Pickering emulsion stabilized by the lipid particles, data reproduced from [190]. Korsmeyer model (below) for sample A (**a**–**h**) cal image and (B) SM (**a′**–**h′**) image, data reproduced from [191].

Orally administered bioactive compounds are prone to rapid degradation. Therefore, stable and robust co-delivery vehicles are needed to protect the active substances from degradation in the orogastric environment and to facilitate their eventual release and absorption in the small intestine. However, differences in the structure and encapsulation of the active substance by the co-delivery vehicle can significantly affect the release of the active substance (Figure 3). Release kinetic studies reveal the effects of carrier material, structure, and environmental factors (e.g., pH, enzymes, temperature) on release behavior by analyzing the rate, pattern, and triggering conditions of active substance release from carriers [185]. By using release kinetic models (e.g., diffusion-controlled, solvation-controlled, or swelling-controlled), the release behavior of the active substance can be predicted, thus guiding the controlled release design of the carrier [186]. In addition, release kinetic studies help to balance the relationship between release rate and bioavailability. Too fast a release may lead to localized toxicity (e.g., damage to normal tissues by adriamycin), whereas too slow a release may reduce efficacy. In co-delivery systems, release kinetics is also required to harmonize the release timing of different actives to achieve optimal therapeutic efficacy. The kinetic equations commonly used to characterize the release of active substances are zero-order, first-order, Weibull, Hixson–Crowell, Higuchi, Korsmeyer–Peppas, and other model equations [187]. The equation fitted when the rate of substance release is independent of the substance concentration is the zero-order kinetic equation, while the equation fitted when the rate of substance release is positively related to the substance concentration is the first-order kinetic equation. Dissolution degree, as an important parameter of substance release, directly affects the bioavailability and clinical efficacy of the substance. The Weibull model has a wide range of applications in dissolution studies and can effectively describe the dissolution process of different substances in vitro and in vivo. The Weibull model is highly adaptable, and the distribution can effectively fit different types of dissolution patterns [188]. The Hixson–Crowell model is used to characterize the rate of drug release over time in solid drug formulations. It should be noted that the Hixson–Crowell model is empirical and needs to be analyzed in conjunction with other models. The Higuchi model is also used as an empirical model to describe the release kinetics of a given solute in a colloidal, porous medium. The principle of this equation is the diffusion process that occurs in the boundary layer of a solid, which results in the outward diffusion and gradual release of solute molecules. The Korsmeyer–Peppas model is a model that describes the rate of release of a drug as a function proportional to a power function of time and the exponential n of the drug content [189]. The model applies to all types of solid formulations to study slow and controlled release. Consequently, this paper compares the release kinetic studies of several co-delivery carriers and further analyzes the reasons for the differences in the release of active substances in co-delivery carriers at the molecular docking level.

The co-release of co-encapsulated Sudan III and DMP from SLN1-stabilized water-in-oil emulsions prepared using sunflower oil as a continuous phase was investigated by Georgia I. Sakellari et al. [190]. The variation in the release rate of Sudan III is attributed to the active enrichment of the active substance near the particle shell and the large surface area of the nanoparticles. The slow release at a later stage may be the result of the diffusion of the remaining active substance in the inner regions of the lipid matrix. The release of Sudan III is best described by the Weibull model. Sodium alginate-based microparticles (MpB) and microparticles consisting of (Ethyl cellulose (EC) + polyethylene glycol (PEG)) (MpP) mixtures were successfully prepared by Gonçalves et al. [32] using the spray-drying technique for the co-encapsulation and co-administration of RA, curcumin, and/or resveratrol. When fitting the Weibull model to the controlled release curve, a correlation coefficient of 0.991 was obtained for the MpP. Of all the particulate formulations, the Weibull model is best suited to ensure a complete fit to the controlled release profiles of all bioactive compounds obtained from MpB and MpP. The Weibull model is best suited to describe the release of bioactive compounds from matrix-based particles, which is related to the typical morphology obtained by spray-drying techniques [192]. Liu et al. [83] understood the release of βC from L-βC and L-VC-βC by fitting a release kinetic model, and used the Korsmeyer–Peppas model to describe the mechanism of βC release from both liposomes in the small intestine. The Korsmeyr–Peppas model is best suited to describe the release of βC from the intestinal phase of L-βC and L-VC-βC. The release of active ingredients is dominated by Fickian diffusion. The rate of release of the active substance during gastrointestinal digestion suggests that liposomes protect the active substance from damage in the stomach and release it into the small intestine, where it is absorbed. The in vitro release kinetics of βC from both liposomes suggest that the release mechanism is dominated by Fickian diffusion. The release patterns of Cur and Res from digested hydrogels are investigated by in vitro simulation by Yao et al. [109], and it was found that the final release of Res is twice as much as that of Cur, suggesting that Res is better suited to be captured in hydrogel networks. Overall, there are significant differences between Cur and Res encapsulation, including release amount, release kinetics, encapsulation time, and complexation mechanism, and their differences in molecular size and weight appear to be responsible for the observed variations. The Cur complex is found to be most compatible with the Korsmeyer–Peppas model, and the two stages of Res release best fit the Makoid–Banakar and Korsmeyer–Peppas models, respectively. Release kinetics may also be affected by water uptake and swelling of the IC matrix, and release may be caused by the relaxation of the keratan gum network structure. The specific release kinetics between Cur and resveratrol imply that there are different molecular affinities between the IC chains and these polyphenols, which may be influenced by the size and polarity of the encapsulated core material.

Under the complex physiological environment in vivo, delivery carriers may experience unintended early release or incomplete release, affecting the application effect. At the same time, the differences in physiological environments among different individuals may lead to inconsistent release kinetics. Therefore, it is necessary to thoroughly study the release mechanism of carriers in complex environments in vivo and ex vivo, and to develop multi-responsive carriers to improve the precision and controllability of release. To further investigate the mechanism of release of bioactive substances from co-delivery carriers, many studies have used molecular docking techniques to investigate the binding modes and interaction forces between actives and carriers. To further explore the mechanism of interaction between riboflavin, rhodopsin, and glucan-protein, the binding sites and binding forces were analyzed using molecular docking. The results showed the existence of conventional hydrogen bonding between riboflavin (RIB) and Lys-261 and -265 of glutenin; carbon–hydrogen bonding between RIB and Glu-126 and Asp-131 of glutenin; and hydrogen bonding between rhodopsin (RHE) and Arg-366, -397, -499, and Ala-492 of glutenin. RHE is conventionally hydrogen bonded to Arg-366, -397, -499, and Ala-492 of gluten. In addition, RIB can bind to dextran via conventional hydrogen, ϰ-stack, and π-σ bonds. RHE can bind to dextran via conventional hydrogen and π-stack bonds. The higher binding energy of dextran to the actives compared to the binding energy of the actives to gluten indicates that the actives mainly tend to bind to gluten. Riboflavin and rhodopsin, and dextran–glutenin, can form spontaneous binding, and hydrogen bonding plays an important role in the combination [193]. Both procyanidin B2 (PB2) and dihydromyricetin (DMY) were bound to the outer surface site of β-lactoglobulin (β-LG). It is worth noting that the Trp-19 residue in β-LG may be responsible for the efficient fluorescence quenching of β-LG by PB2 and DMY. Two hydrogen bonds were formed between the PB2 molecule and β-LG. Although the binding sites of PB2 and DMY on β-LG were similar, the different number and distance of hydrogen bonds gave DMY a stronger binding affinity. Therefore, the results of molecular docking showed that hydrogen bonding played a major role in the binding of the two ligands to β-LG, which further confirmed the fluorescence analysis [43]. In addition to the use of molecular docking-assisted kinetics to further elucidate the release of actives from co-delivery vehicles and in addition to fluorescence resonance energy transfer techniques [194], stable isotope tracer methods [195], bio-imaging techniques [196], and a combination of in vitro simulations and in vivo experiments [197] can be used to assist in the elucidation.

**Figure 4:**
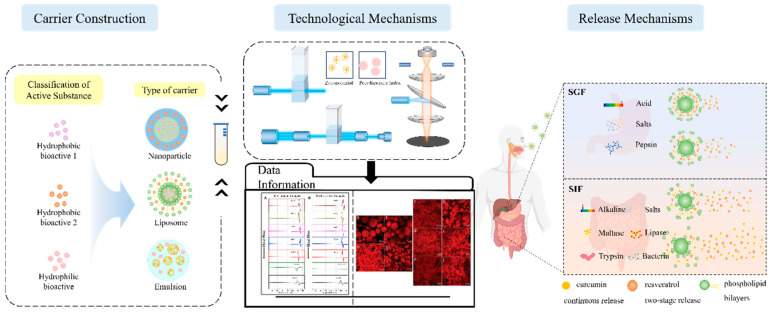
Schematic diagram of co-delivery vector construction–technology mechanism–release. (**A**,**B**) Data Information represents the DSC results plot and (**a**–**j**) represents the CLSM results plot. Data reproduced from [109,110].

Figure 4 plots the process from the carrier construction–technological mechanism to the release mechanism. The rate of release of nanoparticles is influenced by factors such as their particle size, surface properties, and solubility of the substance. Smaller particle sizes usually contribute to faster release, often consistent with zero- or first-level kinetics. And when liposomes released two bioactives simultaneously, the researchers found that the Korsmeyer–Peppas model was more in line with the release characteristics of the bioactives, which are dominated by Fickian diffusion [189]. In addition, liposomes can protect the active substances from degradation during the gastric digestion stage to a certain extent, and successfully release and absorb them during the intestinal digestion process. The crosslink density and network structure of hydrogels have a significant effect on drug release, which is more in line with the Higuchi equation or the Korsmeyer–Peppas equation. Hydrogels with high crosslink density usually have slower drug release rates [198]. However, the Weibull model can fit perfectly with the controlled release profiles of all bioactive substances, mainly due to the use of the spray drying technique. The significant differences in the release kinetics of different bioactive substances in the hydrogel system may be related to the magnitude of the molecular weight of the actives and the differences in the binding force between the bioactive substances and the hydrophilic gel matrix. In addition, although single active substance delivery systems can achieve stable controlled release, they cannot fully utilize the synergistic effect of multiple components. In contrast, the co-delivery system has significant synergistic effects, but the compatibility of multiple components needs to be solved, and the carrier material needs to take into account the physicochemical properties of different components. To optimize the delivery system, microfluidic technology can be used to enhance the batch consistency of nanocarriers and avoid the problem of uneven particle size distribution in the traditional process. In addition, the establishment of an in vitro–in vivo correlation model can effectively reduce experimental errors and improve the reliability of this study. However, there are still shortcomings in the existing technologies. For example, the chemical stability and release competition mechanism between components in the co-embedding system need to be further analyzed. In addition, individualized carrier design based on multi-omics data is still in the preliminary stage and is not yet mature. Future research directions can be optimized by optimizing the carrier materials, improving the preparation process, and studying the release mechanism in depth, which is expected to promote the clinical application of precision drug and nutrient delivery systems and provide more efficient technical support for personalized medicine.

## 4. Application of Co-Delivery Carriers

**Figure 5:**
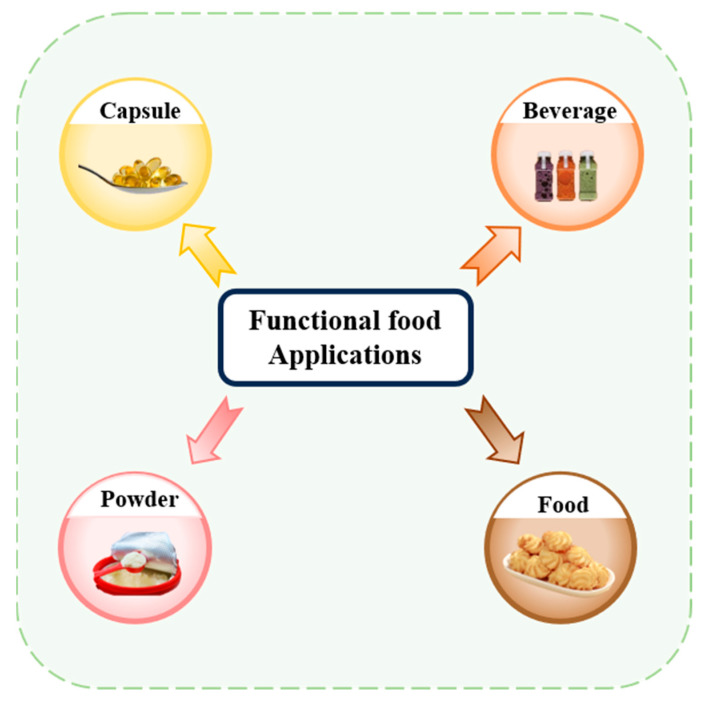
Intelligent co-delivery technology application schematic.

With the rapid growth of food technology and consumption levels, the demand for functional foods has increased. Compared to functional foods with a single active substance, functional foods with multiple active substances co-delivered in one system have received widespread attention. Due to the synergistic effect of the co-delivery system on the bioactivity of the active substances, multiple bioactivities are exerted, which provide excellent health benefits and wellness effects. Functional foods are closely related to precision nutrition, which refers to the optimization of health and the prevention, management, and treatment of disease through personalized nutritional support during nutritional interventions, and is also a consideration in the design of co-delivery vehicles [69]. Therefore, researchers and scholars have developed many application systems containing co-delivery carriers for active substances. In the current research, common delivery vehicles mainly include emulsions, nanoliposomes, nanoparticles, gels (hydrogels, oleogels, and emulsion gels), and microcapsules. Based on the encapsulation of multiple active substances (polyphenols, vitamins, bacteria, and proteins, etc.) by co-delivery vehicles, bioactive synergism can be used in a wide range of applications. In addition to this, the specificity of the co-delivery vehicle itself provides the basis for its application. Currently, the common applications of co-delivery systems are mainly in the field of food (Figure 5) [199,200,201,202,203,204,205,206,207].

Nanoparticles co-loaded with proanthocyanidins and Cur had a high encapsulation rate (>95%) with a synergistic antioxidant effect (78.4%), which has significant potential in functional food applications [21]. The bigel system has a slow release in the stomach and mouth and a rapid release in the intestines to effectively inhibit colon cancer and achieve precision nutrition [110]. The multilayered structured nanoparticles prepared by Wang et al. enable programmed release (in the small intestine and colon, respectively), which can contribute to the development of functional foods that benefit intestinal health [64]. Chitosan liposomes co-encapsulating glutathione and edible phenolic acids have a strong synergistic antioxidant effect and can be applied to antioxidant-type functional foods [5]. Plant-based emulsions are a current research hotspot and are popular due to their health and environmental friendliness, wide range of applicability, and unique flavor and texture [208]. The co-encapsulation of curcumin and catechin in olive oil-based double emulsions has been applied in the field of nutrient-fortified milks with good stability and a wide range of applications [209]. Microencapsulated powders were prepared by Huang et al. [210] using spray drying, which produced powders with excellent antioxidant properties and high retention of bioactives (>96%). These powders could be made into capsules for use in health foods. Microcapsules containing xanthan gum are expected to be used in the development of functional foods involving thermal processing, such as soups, hot beverages, and baked goods. This excellent preservation is attributed to the semi-crystalline nature of xanthan gum, which restricts the mobility of molecules and inhibits the transfer of heat and oxygen [74]. The co-encapsulation of blackberry juice and probiotics in a biopolymeric matrix can be used in yogurts and juices with the benefit of regulating gut health [211]. Excessive intake of fat was a risk factor for chronic diseases like obesity, diabetes, and metabolic syndrome. However, removing fat from foods was challenging because of its critical role in providing desirable appearances, textures, mouthfeels, and flavors [212]. Co-delivery vehicles such as emulsions and oleogels can be applied to solid fat substitutes to provide a similar texture and mouthfeel, while also being able to reduce their fat content. Pickering emulsion was applied to co-deliver EGCG and lycopene to induce synergistic hypolipidemic effects and significantly reduce adipose tissue mass in mice [35]. Novel emulsion gel textural properties stabilized with beeswax can be used to replace cheese, expanding its application in food [34].

Currently, the co-delivery system based on multi-component synergy has become an important direction in the development of functional food products. This technology significantly improves the bioavailability and functional synergy of active ingredients through scientific compounding and delivery carrier innovation [151]. For example, BY-HEALTH’s lutein ester and the β-carotene combination capsule realize the synergistic delivery of eye nutrients through microencapsulation technology. Uncle Matt’s developed a functional drink with black pepper ingredients, which adopts bioavailability enhancement technology to improve the absorption rate of Cur. SmartyPants innovatively developed polysaccharide–protein composite carrier technology to realize the synergistic effect of Omega-3, DHA, and other nutrients in the eye area. These innovations demonstrate the unique advantages of co-delivery systems in enhancing bioavailability and achieving synergistic effects. However, the clinical delivery of bioactive ingredient delivery systems still faces multidimensional challenges. At the technical level, there are data gaps in the biosafety assessment and long-term toxicity studies of delivery vehicles, which make it difficult to meet the stringent approval requirements of food standards [213]. The Food and Drug Administration allows companies to self-certify the safety of nano-delivery materials based on them being Generally Recognized as Safe (GRAS), but the burden of proof is on them. If they are not GRAS-certified, they are required to report this according to the food additive approval process. The European Food Safety Authority released the “Guidance on Risk Assessment for Nanotechnology Applications” in 2021, which explicitly requires the systematic assessment of nanomaterials, covering key steps such as physicochemical characterization (e.g., particle size, stability), hazard identification and characterization, and exposure and risk analysis to confirm their safety in food. Neither agency has issued specific detailed standards for nanodelivery materials, and the risk assessment framework still relies on existing general rules combined with case-by-case reviews. At the industrialization level, the cost control and scale-up process of the novel carriers are still in their infancy, and the lack of clarity on the applicability of regulations constrains their commercialization. To this end, there is an urgent need to establish an interdisciplinary synergistic innovation mechanism. In the field of food and agriculture, it is necessary to build a database of ingredient–carrier–substrate compatibility and formulate specifications for grading and classifying management.

## 5. Conclusions and Future Trends

Co-delivery systems are commonly used to encapsulate more than two different bioactive substances as they enable bioactive synergism, stability, and bioavailability enhancement. In this review, we summarized the properties, strengths, and weaknesses of different types of bioactive substances and the improved effects of co-delivery systems. In addition, recent developments in co-delivery systems, including delivery vehicles such as emulsions, nanoparticles, and liposomes, among others, are discussed, and their improved effects on antioxidant activity, stability, and bioavailability are compared. And the means of characterization of the co-delivery carriers were compared. More importantly, this study also investigated the differences in the simulated in vitro release kinetics of the active substances and elucidated their intrinsic mechanisms by molecular docking techniques. Finally, the prospects for the application of co-delivery carriers in functional foods were summarized. In conclusion, this study can provide some theoretical references for the field of co-delivery and promote the development of rational and efficient co-delivery system construction.

The development and application of co-delivery technologies still face multiple challenges in many areas. The design of stimuli-responsive carriers requires a balance between responsiveness and stability. While chemical coupling can enhance carrier stability and confer targeted responsiveness, the complex environment of specific stimulus conditions (e.g., redox, pH) can lead to stability challenges. For example, disulfide bonds need to remain inert in the circulation and respond to breakage only in strongly reducing tumor cells. pH-sensitive linkers may result in uncontrolled release due to tumor microenvironmental pH heterogeneity (5.0–7.4). In addition, there is a risk of off-targeting, and the distribution of stimulus signals in normal tissues may trigger non-specific release. Moreover, its biocompatibility requirements are strict. The carrier material should avoid immune reaction and realize controlled degradation, and some of the degradation products of synthetic polymers have toxicity potential. Finally, individualized differences affect efficacy, and individual fluctuations in tumor microenvironmental parameters can lead to differences in release kinetics. Stimuli-responsive design has a bright future in the field of delivery, but in-depth research and optimization are needed to balance responsiveness and stability. In order to break through these bottlenecks, we can start from the following aspects in the future: (1) With the help of computer-aided design and high-throughput screening technology, we can carry out precise design for the carrier of co-carrying active substances, so as to enhance the relevance and efficiency of the technology; (2) we can research and develop highly efficient and scalable production of the preparation process to improve the stability of the quality of the carrier, and at the same time reduce the cost, so as to lay a foundation for the industrialization of the application of the technology; (3) we can establish innovative characterization techniques to achieve interference-free dynamic tracking of the interaction between active substances and carriers and the release process, and to enhance the universality and reliability of the technology; (4) we can conduct an in-depth study of the release mechanism of carriers in the body’s complex environment, and the design of a personalized release system, to meet the needs of precision medicine; and (5) we can strengthen interdisciplinary cooperation and joint solutions for transformation from the laboratory to the actual application of the technology, and to promote its clinical application and industrialization. Through the systematic integration of the above strategies, co-administration technology is expected to break through the bottleneck of existing technologies and provide more efficient technical support for precision delivery and personalized medicine.

## Figures and Tables

**Table 1:** Comparison of functional properties of bioactive substances.

Active Substance	Structural Formula	Characteristic	Function	Bioavailability	Stability	References
EGCG	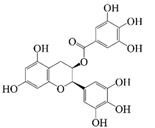	Hydrophilic	Anticancer	--	Thermal stability and UV light stability	[19]
Tea polyphenols	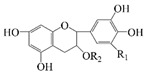	Hydrophilic	Antioxidant and anticarcinogenic	--	Storage stability	[20]
Procyanidins	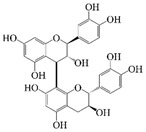	Hydrophilic	Antibiotic, antioxidant, and anti-inflammatory	--	--	[21]
Ellagic acid	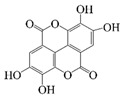	Hydrophobic	Antidiabetic, antioxidant, and anti-inflammatory	--	Storage stability	[22]
Resveratrol	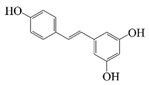	Hydrophobic	Anti-inflammatory, antioxidant, anticancer, liver-protective	--	pH stability, salt stability, and thermal stability	[23]
Quercetin	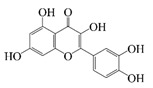	Hydrophobic	Antioxidant, anti-inflammatory and antibacterial activities, improvement of cardiovascular health	--	Storage stability	[24]
Curcumin	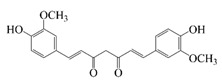	Hydrophobic	Reduce blood lipid levels, antitumor activity, anti-inflammatory activity, antioxidant activity, and inhibition of Alzheimer’s disease	87.3 ± 2.8%	pH stability	[25]
VCAM-1 siRNA	--	Hydrophilic	anti-inflammatory	--	--	[26]
Berberine	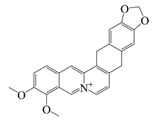	Hydrophobic	Anticholinergic, antihypertensive, antibacterial, anti-inflammatory, and antioxidative	--	Storage stability	[27]
Paclitaxel	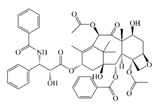	Hydrophobic	Glioma	--	--	[28]
Lactobacillus casei	--	Hydrophilic	Maintain gut balance by shaping gut microbiota and intestinal barrier	--	Thermal stability, and storage stability	[29]
Probiotics	--	--	Regulate the intestinal microbiota	--	Photochemical stability and storage stability	[30]
Vitamin C	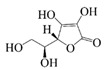	Hydrophilic	Antioxidant	--	Storage stability	[31]
Retinoic acid	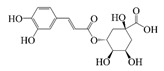	Hydrophobic	Acute promyelocytic leukemia	--	--	[32]
Vitamin B12	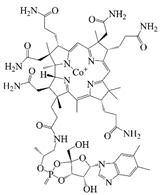	Hydrophobic	--	99%	Storage stability	[33]
Vitamin D3	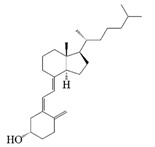	Hydrophobic	Bone development and bone health	97%	Storage stability	[33]
Astaxanthin	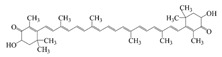	Hydrophobic	Anti-oxidative, anti-inflammatory, and anti-apoptotic	43.2 ± 0.3%	Freeze–thaw stability	[31,34]
Lycopene	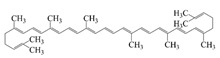	Hydrophobic	Antioxidant and cholesterol metabolism	--	Storage stability	[35]
β-Carotene	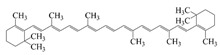	Hydrophobic	Antioxidant	--	Storage stability	[31]
Cinnamon oleoresin	--	Hydrophobic	Antimicrobial	--	Storage stability	[36]
Cinnamaldehyde essential oil	--	Hydrophobic	Antimicrobial	--	Storage stability	[37]
Tuna fish oil	--	Hydrophobic	Health-promoting benefits and disorder-prevention attributes	--	Oxidative stability	[38]
Coenzyme Q10	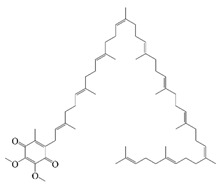	Hydrophobic	--	--	Storage stability	[20]
Phycocyanin	--	Hydrophilic	Hepatoprotective, neuroprotective, and antitumor	41.6 ± 0.5%	Freeze–thaw stability	[31,34]
Glutathione (γ-L-glutamyl-l-cysteinylglycine	--	Hydrophilic	Antioxidant	--	Storage stability	[5]
Melittin	--	Hydrophilic	Glioma	--	--	[28]
Nisin	--	--	Antimicrobial	--	--	[39]

Note: EGCG: Epigallocatechin gallate; Cur: Curcumin; Bioavailability: The degree to which a substance, after being ingested, is absorbed through the gastrointestinal tract into the blood circulatory system and then utilized by tissues and organs. A “--” in the table indicates that no relevant data are available or that data were not captured.

**Table 2:** Summary of co-loaded actives in different delivery vehicles.

Carrier Type	Active Substances	Materials	Preparation Methods	Encapsulation Efficiency	Bioavailability	Precision Nutrition	References
O/W emulsion	α-tocopherol and resveratrol	Sodium caseinate and sunflower oil	High-speed blend, high-pressure homogenize	92–96%	40%, 90%	--	[7]
Pickering emulsion	EGCG and Cur	MCT, WPI, and d-lactose	High-speed homogenize	--	--	--	[49]
Pickering emulsion	EGCG and lycopene	Soybean oil, PGPR, and bacterial cellulose	Magnetic stir, high-speed homogenize	--	--	Hypolipidemic	[35]
Pickering emulsion	Betanin and Cur	Gelatin and PGPR	--	65.3%, 84.1%	42.7%, 53.5%	Antitumor	[90]
Pickering emulsions	Cur and β-carotene	Zein, MCT, and tea saponin	Microfluidization	97.58 ± 0.12%, 96.49 ± 0.46%	75.33 ± 5.11%, 19.46 ± 0.59%	--	[91]
W/O/W emulsion	Vitamin C and β-carotene	Soybean oil, PGPR, and sipunculus nudus	Magnetic stir, high-speed homogenize	91.2%, 99.8%	--	--	[31]
W/O/W emulsion	Insulin and quercetin	Tween 80 and soybean oil	Stir, ultra-turrax, shear	95.7%, 93.4%	52.33%, 58.7%	Diabetes	[92]
W/O/W emulsion	Cur and catechin	Olive oil	Two-step emulsification method	88–97%	54%	--	[9]
W/O/W emulsion	Cur and EGCG	Protein fibril-cellulose, cellulose nanocrystals, and PGPR	Two-step emulsification method	98.0 ± 1.2%, 89.7 ± 0.3%	67.8%, 68.9%	--	[52]
Nanoliposome	Coenzyme Q10 and α-lipoic acid	CS, Soy phosphatidylcholine, agar, cholesterol, and Tween 80	Magnetic stir, inject	--	--	--	[6]
Nanoliposome	Reduced glutathione and caffeic acid	CS, soybean lecithin, and cholesterol	Two-step emulsification procedure	61.32%, 68.92%	--	--	[5]
Nanoliposome	Vitamin C and β-carotene	Egg yolk phosphatidylcholine and cholesterol	Ethanol injection method	77.90 ± 1.92%, 97.91 ± 0.20%	--	--	[83]
Nanoliposome	Cur and resveratrol	Egg yolk phosphatidylcholine	Thin-film evaporation method	80.42 ± 2.12%	--	--	[70]
Nanoliposome	Coenzyme Q10 and Cur	CS	--	98.5%	--	--	[86]
Nanoliposome	Vitamin B2, vitamin E, and β-carotene	Lecithin from soybean and cholesterol	Thin-layer dispersion method	--	--	--	[82]
Nanoliposome	Trans-resveratrol and naringenin	Egg yolk phosphatidylcholine and Tween 80	Thin-film evaporation method	77.72 ± 2.42%, 61.98 ± 1.68%	--	Antioxidant	[93]
Nanoliposome	Shikonin and anthracyclines	Hydrogenated soybean phosphatidylcholine, cholesterol, and DSPE-PEG2000	Thin-film hydration method	95%	--	Antitumor	[94]
Nanoliposome	Lupulon and xanthohumol	Egg yolk lecithin	Sonication method	81.7%, 97.13%	--	Antimicrobial	[95]
Nanoparticle	Cur and serrati peptidase	BSA	Desolvation method	80%, 8.4%	--	Anticancer	[96]
Nanoparticle	Cur and EGCG	HA and mPEG5000-NHS	Stir	46.01 ± 1.96, 67.76 ± 6.67%	--	Anticancer	[97]
Nanoparticle	Cur and PC	SPI	Flash nanoprecipitation	95%	--	--	[21]
Nanoparticle	α-Tocopherol, resveratrol, and EGCG	BSA	Mix	--	--	Antioxidant	[98]
Nanoparticle	Cur and EGCG	Yeast protein	Stir	--	--	--	[99]
Nanoparticle	FUC and Cur	Gliadin, CS hydrochloride, and CMK	Stir	96.3%, 72.8%	--	Colon-targeted	[64]
Nanoparticle	Ellagic acid and anti-inflammatory peptide	CS, TPP, Dex, and LPS	Ionic gelation method	--	--	Anti-inflammatory	[22]
Nanoparticle	Resveratrol and vitamin D3	Olive oil	pH shift combined with heat treatment process	86.74%, 53.24%	81.2%, 93.2%	--	[100]
Nanoparticle	Procyanidin B_2_, dihydromyricetin	β-lactoglobulin	--	--	--	--	[43]
Nanoparticle	Egg white-derived peptides and Cur	β-Cyclodextrin and HTCC	Spontaneous self-assembly	94.7–98.9%	--	--	[101]
Nanoparticle	Cur and doxorubicin hydrochloride	Tyrosine, PASP, HA, and EDA	--	Loading capacity: 50.9 ± 4.3%, 26.0 ± 1.9%	--	Anticancer	[102]
Nanoparticle	Cur and berberine	Zein and CS	Stir	75%, 60%	--	--	[27]
Microcapsule	Novel bacteriocin lactococcin036019, and vitamin C	S. Aureus cmcc 26003	High-speed disperse, spray-dry	56.18%	--	Antibacterial	[103]
Microcapsule	Cur, quercetin, tea polyphenols, and lyophilized lactobacillus casei	Zein, CS, and rutin	Anti-solvent process, coacervate,	68.44 ± 1.3%%, 57.35%, 58.13%	--	--	[29]
Microcapsule	RA, Cur, and resveratrol	Alginic acid sodium salt and coconut oil	Spray-dry	96 ± 5%, 92 ± 4%, 93 ± 4%	--	--	[32]
Microcapsule	Xylitol and menthol	Pork gelatin type B, gum Arabic, corn oil, and PGPR	Spray-dry	--	--	--	[104]
Microcapsule	Cur and pancreatic beta cells	Alginate	Jet-break regime of the syringe pump	--	--	Diabetes	[105]
Microcapsule	Resveratrol and fish oil	Whey protein, gum Arabic, and transglutaminase	Spray-dry	84–88%	--	--	[106]
Microcapsule	Paprika and cinnamon oleoresin	WPI and maltodextrin	Spray-dry	83%	--	--	[36]
Microcapsule	Resveratrol and piperine	Hydroxypropyl β-cyclodextrin	Spray-dry	--	--	--	[107]
Microcapsule	Anthocyanins and α-tocopherol	Gum Arabic	Spray-dry	--	--	--	[59]
Emulsion gels	Astaxanthin and phycocyanin	Beeswax, gelatin, sodium caseinate, sodium alginate, low acylated gellan gum, and corn oil	Fast digital high-speed shear, homogenize	96.4 ± 0.9%; 94.9 ± 2.1%	43.2 ± 0.3%; 41.6 ± 0.5%	--	[34]
Emulsion gels	Quercetin and EGCG	PGPR, corn oil, wheat gluten, and gelatin	Antisolvent precipitation method, high shear mix	65.5%, 97.2%	48.4%, 49%	--	[55]
Hydrogel	EGCG and Cur	Defatted soy flour	Heat followed by ice water bath	97.71%, 91.02%	--	Anticancer	[19]
Hydrogel	Cur and resveratrol	Zein, gelatin, gellan gum, and polyethylene glycol	Stir under heating followed by cooling at room temperature	68.33%, 83.39%	82%, 79%	--	[108]
Hydrogel	Cur and resveratrol	Iota-carrageenan	--	--	--	--	[109]
Bigel	EGCG and Cur	Corn oil, gelatin, and Tween 20	Stir	--	--	--	[56]
Bigel	EGCG and Cur	Corn oil, κ-carrageenan, monoglycerides, Tween 20, and PGPR	Stir	--	--	Colon adenocarcinoma	[110]
Oleogel	Cur and resveratrol	Gelatin, gellan gum, and MCT	Stir	80.06 ± 1.37%, 86.41 ± 2.28%	50.08 ± 1.27%, 89.19 ± 0.89%	--	[65]
Nanogel	Cur and doxorubicin	NIPAAm, TEGDMA, PPS, and PVA	Free radical polymerization methods, stir	96%, 98%	--	Colon cancer	[111]
Lipodisk	Paclitaxel and melittin	POPC, DSPE-PEG2000, and cholesterol	Film hydration adsorption method	--	--	Anti-glioma effect	[28]
β-cyclodextrin	Trans-ferulic acid and gallic acid	Hydroxypropyl-β-cyclodextrin	Spray-dry	89.22%, 68.06%	--	--	[112]
β-cyclodextrin	Resveratrol and piperine	Hydroxypropyl-β-cyclodextrin	Spray-dry	--	--	--	[107]
Micelle	Doxorubicin and paclitaxe	Folate, oxidized dextra, NHS	Schiff’s base reaction	--	--	Anticancer	[113]
Micelle	Doxorubicin and sipd-l1	CS and HA	Nanoprecipitation technique	91.3 ± 2.1%	--	Antitumor	[114]
Hydrogel beads	EGCG and quercetin	Soybean protein isolate, PGPR, sodium alginate	Stir, inject	98.75 ± 0.04%, 96.46 ± 0.76%	--	IBD	[115]
Ca(ii)-alginate beads	Betalains, phenolic compounds	Egg albumin, whey protein	Ionic gelation	70%	--	--	[116]
Copolymer	Polycaprolactone, poly (2-ethyl 2-oxazoline)	Starch	--	--	--	Anticancer	[117]
Niosome	Cur and boswellic acids	CS	--	--	79.02 ± 0.13%, 81 ± 0.10%	--	[118]

Note: INS: Insulin; Q: Quercetin; EGCG: Epigallocatechin gallate; Cur: Curcumin; MCT: Medium-chain triglyceride; WPI: Whey protein isolates; ChA: Chlorogenic acid; CS: Chitosan; BSA: Bovine serum albumin; HA: Hyaluronic acid; PC: Procyanidins; PGPR: Polyglycerol polyricinoleate; FUC: Fucoxanthin; TPP: Tripolyphosphate, Dex: Dexamethasone; LPS: Lipopolysaccharide; RA: retinoic acid; DSPE-PEG2000: 2-distearoyl-snglycero-3-phosphoethanolamine-n-[methyl (polyethylene glycol)-2000; mPEG5000-NHS: methoxyl PEG succinimidyl ester; CMK: Carboxymethyl konjac glucomannan; cRGD-PEG: cRGD-poly(ethylene glycol); PLGA: poly(lactic-co-glycolic acid); PEI: polyethylenimine; HTCC: N-[(2-hydroxy-3-trimethyl ammonium) propyl] chitosan; PASP: poly(aspartic acid); EDA: ethylenediamine; NIPAAm: N-Isopropylacrylamide; TEGDMA: tetraethylene glycol dimethacrylate; PPS: potassium persulfate; PVA: polyvinyl alcohol; POPC: 1-palmitoyl-2-oleoyl-sn-glycero-3-phosphocho-line; NHS: N-Hydroxysuccinimide; Bioavailability: The degree to which a substance, after being ingested, is absorbed through the gastrointestinal tract into the blood circulatory system and then utilized by tissues and organs. A “--” in the table indicates that no relevant data are available or that data were not captured.

## Data Availability

The original contributions presented in this study are included in the article. Further inquiries can be directed to the corresponding author.
